# The cGAS–STING pathway drives type I IFN immunopathology in COVID-19

**DOI:** 10.1038/s41586-022-04421-w

**Published:** 2022-01-19

**Authors:** Jeremy Di Domizio, Muhammet F. Gulen, Fanny Saidoune, Vivek V. Thacker, Ahmad Yatim, Kunal Sharma, Théo Nass, Emmanuella Guenova, Martin Schaller, Curdin Conrad, Christine Goepfert, Laurence de Leval, Christophe von Garnier, Sabina Berezowska, Anaëlle Dubois, Michel Gilliet, Andrea Ablasser

**Affiliations:** 1 Department of Dermatology, CHUV University Hospital and University of Lausanne (UNIL), Lausanne, Switzerland; 2 Global Health Institute, Swiss Federal Institute of Technology Lausanne (EPFL), Lausanne, Switzerland; 3 University Department of Dermatology, Eberhard Karls University of Tübingen, Tübingen, Germany; 4 Institute of Animal Pathology, COMPATH, University of Bern, Bern, Switzerland; 5 Histology Core Facility, Swiss Federal Institute of Technology Lausanne (EPFL), Lausanne, Switzerland; 6 Institute of Pathology, CHUV University Hospital and University of Lausanne (UNIL), Lausanne, Switzerland; 7 Division of Pulmonology, CHUV University Hospital and University of Lausanne (UNIL), Lausanne, Switzerland; 8 Biological Electron Microscopy Facility, Swiss Federal Institute of Technology Lausanne (EPFL), Lausanne, Switzerland

**Keywords:** Innate immunity, SARS-CoV-2, Infection

## Abstract

COVID-19, which is caused by infection with SARS-CoV-2, is characterized by lung pathology and extrapulmonary complications^1,2^. Type I interferons (IFNs) have an essential role in the pathogenesis of COVID-19 (refs ^3–5^). Although rapid induction of type I IFNs limits virus propagation, a sustained increase in the levels of type I IFNs in the late phase of the infection is associated with aberrant inflammation and poor clinical outcome^5–17^. Here we show that the cyclic GMP-AMP synthase (cGAS)–stimulator of interferon genes (STING) pathway, which controls immunity to cytosolic DNA, is a critical driver of aberrant type I IFN responses in COVID-19 (ref. ^18^). Profiling COVID-19 skin manifestations, we uncover a STING-dependent type I IFN signature that is primarily mediated by macrophages adjacent to areas of endothelial cell damage. Moreover, cGAS–STING activity was detected in lung samples from patients with COVID-19 with prominent tissue destruction, and was associated with type I IFN responses. A lung-on-chip model revealed that, in addition to macrophages, infection with SARS-CoV-2 activates cGAS–STING signalling in endothelial cells through mitochondrial DNA release, which leads to cell death and type I IFN production. In mice, pharmacological inhibition of STING reduces severe lung inflammation induced by SARS-CoV-2 and improves disease outcome. Collectively, our study establishes a mechanistic basis of pathological type I IFN responses in COVID-19 and reveals a principle for the development of host-directed therapeutics.

## Abstract (web summary)

The cGAS–STING pathway has a central role in the pathogenesis of severe COVID-19 by driving the increase in type I interferons that occurs in the later stages of SARS-CoV-2 infection.

## Main

**Fig. 1:**
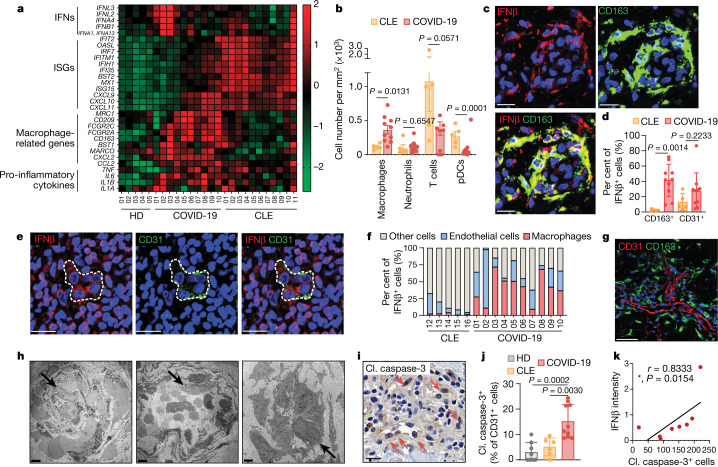
Type I IFN-producing macrophages surround damaged endothelial cells in COVID-19 skin lesions. **a**, Immune gene expression profiles of skin lesions from individuals with COVID-19 (*n* = 10) and individuals with CLE (*n* = 11), and skin from healthy donors (HD; *n* = 5). Unbiased clustering was performed. **b**, Immunohistochemistry quantification of macrophages, neutrophils, plasmacytoid dendritic cells (pDCs) and T cells (stained for CD163, MPO, CD123 and CD3) in CLE (*n* = 5) and COVID-19 (*n* = 10) skin lesions. **c**, Confocal microscopy images of representative COVID-19 skin lesion stained for CD163 (green) and IFNβ (red). Scale bars, 20 μm. **d**, Contribution of CD163^+^ macrophages and CD31^+^ endothelial cells to IFNβ expression in CLE (*n* = 5) and COVID-19 (*n* = 10). **e**, Confocal microscopy images of representative COVID-19 skin lesion stained for CD31 (green) and IFNβ (red). Scale bars, 20 μm. **f**, Proportions of CD163^+^ macrophages, CD31^+^ endothelial cells and other cells among IFNβ-producing cells for each CLE and COVID-19 sample. **g**, Confocal microscopy images of a representative COVID-19 skin sample stained for CD163 (green) and CD31 (red) to depict macrophages and endothelial cells. Scale bar, 50 μm. **h**, Transmission electron microscopy of dermal vessels in purpuro-necrotic (left) and maculopapular (middle) COVID-19 skin lesions, and in healthy skin (right). Arrows show disrupted endothelial cells (COVID-19 skin lesions) and intact endothelial cells (healthy skin). Scale bars, 2 μm (left); 5 μm (middle); 1 μm (right). **i**, Immunohistochemistry for cleaved caspase-3 (cl. caspase-3) in COVID-19 skin lesions (nuclear staining indicated by arrow). Scale bar, 20 μm. **j**, Percentage of CD31^+^ endothelial cells with cleaved caspase-3 staining in healthy skin (*n* = 8) and in CLE (*n* = 6) and COVID-19 (*n* = 10) skin lesions. **k**, Correlation between cleaved-caspase-3-positive nuclei and overall staining intensity of IFNβ measured in COVID-19 skin samples (*n* = 8). Spearman correlation and two-tailed statistical significance were performed. Data are mean ± s.d. (**b**, **d**, **j**). *P* values obtained with two-tailed Student’s *t*-test and one-way ANOVA followed by Tukey’s multiple comparisons test (**b**, **d**, **j**). Source data

**Extended Data Fig. 1:**
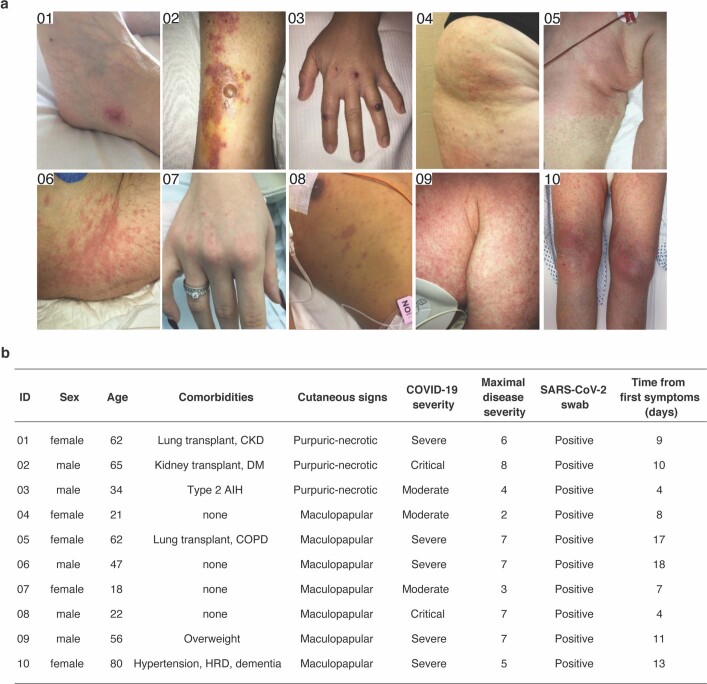
Clinical characteristics of patients with COVID-19 with associated skin manifestations. **a**, Photographs of the skin lesions. **b**, Clinical parameters and demographics of the 10 patients with COVID-19 selected for this study. HRD, Heart Rhythm Disorder; Overweight (BMI > 25 kg/m^2^, but < 30 kg/m^2^); COPD, chronic obstructive pulmonary disease; CKD, chronic kidney disease; DM, diabetes mellitus; AIH, autoimmune hepatitis.

**Extended Data Fig. 2:**
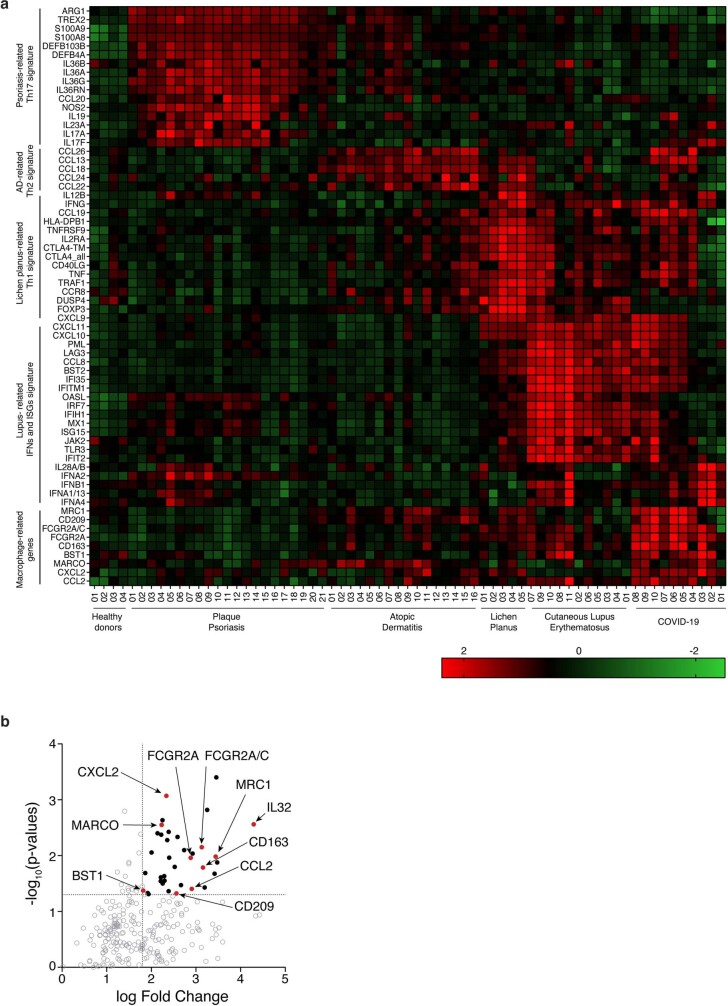
Immune gene expression profiling of COVID-19 skin lesions and common inflammatory skin diseases. **a**, Immune gene expression profiles of patients with lichen planus (*n* = 5), cutaneous lupus erythematosus (CLE, *n* = 10), COVID-19 associated skin lesions (*n* = 10), plaque-type psoriasis (*n* = 21), and atopic dermatitis (AD, *n* = 16) compared to healthy skin (HD, *n* = 4) assessed by NanoString assay. Differentially expressed genes between different pairwise comparisons (i.e. each disease group vs other skin inflammatory diseases) were used to generate disease-related gene signatures. *P* value  <  0.05, and fold change  > 2 were used as cutoffs to choose specific classifiers. **b**, Volcano plot of upregulated genes in COVID-19 compared with CLE skin lesions. Source data

To obtain insight into aberrant immunological processes at the tissue level^19^, we profiled COVID-19-associated skin manifestations from 10 hospitalized patients with moderate-to-severe COVID-19 disease and compared the resultant signatures with those obtained from skin lesions of patients with inflammatory skin diseases^20^ (Extended Data Fig. 1). Transcriptome analysis revealed that COVID-19 profiles clustered with profiles from cutaneous lupus erythematosus (CLE), but separated from signatures of other skin diseases, such as psoriasis, atopic dermatitis and lichen planus (Fig. 1a, Extended Data Fig. 2). Transcriptional similarities between COVID-19 and CLE samples were based on the expression of IFNs (*IFNA2*, *IFNA4*, *IFNA1*, *IFNA13*, *IFNB1*, *IFNL2* and *IFNL3*) in purpuro-necrotic COVID-19 skin lesions and IFN-stimulated genes (ISGs) (*IFIT2*, *BST2*, *IRF7*, *OASL*, *MX1*, *IFITM1*, *IFIT2*, *IFI35*, *IFIH1*, *ISG15*, *CXCL10* and *CXCL9*) in those with a maculo-papular phenotype (Fig. 1a). Notably, both COVID-19 skin phenotypes—but not CLE—showed a marked upregulation of genes related to macrophage function, including macrophage receptors (*CD209*, *CLU*, *MARCO*, *FCGR2A*, *CLEC5A*, *CD163*, *MRC1* and *BST1*), differentiation factors (*IL32*) and monocyte-recruiting chemokines (*CXCL2* and *CCL2*) (Fig. 1a, Extended Data Fig. 2). Pro-inflammatory cytokines (*TNF*, *IL6*, *IL1B* and *IL1A*) were also induced in COVID-19 skin biopsies (Fig. 1a). The immune correlates detected in skin lesions of patients with moderate-to-severe COVID-19 therefore resemble those reported for the lung^8,17^, suggesting a shared mechanism of immunopathology across different organs.

## Vascular damage and type I IFNs in skin lesions

**Extended Data Fig. 3:**
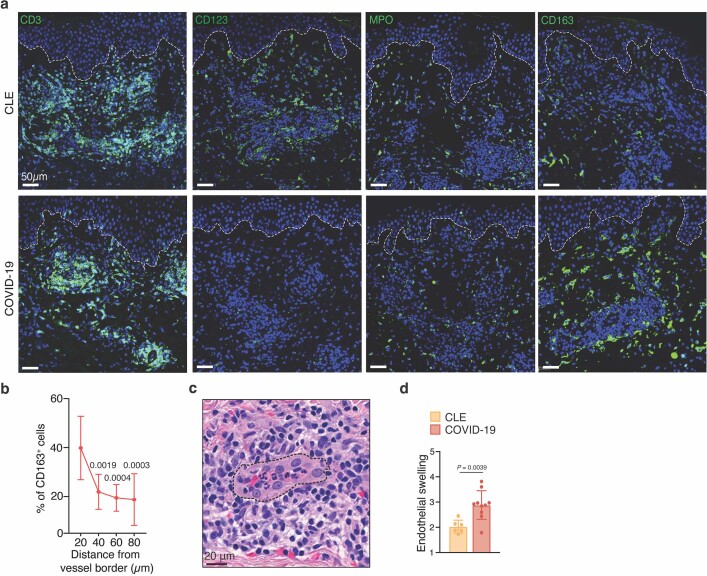
Macrophages accumulate around vessels exhibiting prominent endotheliopathy in COVID-19 skin lesions. **a**, Confocal microscopy images of CD3^+^ T cells, CD123^+^ plasmacytoid dendritic cells, MPO^+^ neutrophils, and CD163^+^ macrophages in skin lesions from CLE (top row) and COVID-19 (bottom row). Images are representative of 10 patients with COVID-19 and 5 patients with CLE. **b**, Percentages of CD163^+^ macrophages present at different distances from blood vessel in COVID-19 skin lesions (*n* = 9). **c**, Representative histopathology image of a dermal blood vessel in COVID-19 skin lesions (H&E stain). Blood vessel, dashed line. **d**, Endothelial cell swelling index, a measure of endotheliopathy, quantified in COVID-19 (*n* = 10) and CLE skin lesions (*n* = 6). *P* values were obtained with one-way ANOVA followed by Tukey’s multiple comparison test (**b**) and two-tailed Student’s *t*-test (**d**). Source data

Next, we comparatively analysed the immune cell composition in skin lesions of patients with COVID-19 and patients with CLE by immunostaining. Consistent with the specific expression of macrophage signature genes, the numbers of CD163^+^ macrophages were higher in COVID-19 skin lesions relative to CLE (Fig. 1b, Extended Data Fig. 3a). By contrast, plasmacytoid dendritic cells—but not macrophages—were enriched in CLE samples, whereas the numbers of neutrophils and T cells were similar in the two conditions (Fig. 1b, Extended Data Fig. 3a). Further examination of macrophages revealed that these cells consistently showed a robust IFNβ response across all samples (Fig. 1c, d, f). Endothelial cells and other cell types also showed a marked IFNβ signal, albeit with a higher degree of inter-sample variability (Fig. 1e, f). We observed that IFNβ-producing macrophages frequently surrounded injured vessels (Fig. 1g, Extended Data Fig. 3b), a well-recognized pathophysiological feature in COVID-19 (refs. ^21,22^). Accordingly, we found several characteristics of endotheliopathy in COVID-19 skin lesions, including endothelial cell swelling (Extended Data Fig. 3c, d), disruption of endothelial cell integrity (Fig. 1h) and nuclear accumulation of cleaved caspase-3 (Fig. 1i, j), a marker for cell death. Notably, the relative amounts of cleaved caspase-3 significantly correlated with levels of IFNβ (Fig. 1k).

## Activation of STING in skin and lung pathology

**Fig. 2:**
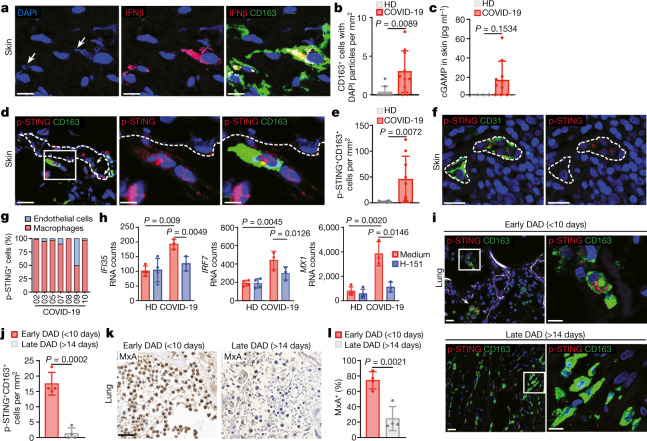
cGAS–STING-dependent type I IFN signature in COVID-19 skin and lung pathology. **a**, Confocal microscopy images of a representative COVID-19 skin sample stained for CD163 (green), *IFNB1* mRNA (red) and DNA (blue). Scale bars, 10 μm. Arrows indicate cytosolic DNA particles. **b**, Quantification of CD163^+^ macrophages containing cytosolic DNA particles in COVID-19 skin lesions (*n* = 10) and in healthy skin (*n* = 9). **c**, Quantification of cGAMP in lysates of COVID-19 skin lesions (*n* = 10) and healthy skin (*n* = 3). **d**, Confocal microscopy images of a representative COVID-19 skin sample stained for CD163 (green) and p-STING (red). Blood vessels, dashed line. Arrows show p-STING^+^ endothelial cells. Scale bars, 20 μm (left); 5 μm (right two images). **e**, Quantification of p-STING^+^ macrophages in COVID-19 skin lesions (*n* = 10) and in healthy skin (*n* = 10). **f**, Confocal microscopy images of a representative COVID-19 skin sample stained for CD31 (green) and p-STING (red). Blood vessels, dashed line. Scale bars, 20 μm. **g**, Proportions of CD163^+^ macrophages and CD31^+^ endothelial cells among p-STING^+^ cells in COVID-19 skin lesions (*n* = 7). **h**, Expression of ISGs (*IFI35*, *IRF7* and *MX1*) in cultured healthy skin (*n* = 3) and COVID-19 skin explants (*n* = 3), treated or not with H-151. **i**, Confocal microscopy images of representative post-mortem lungs with early (fewer than 10 days; left) or late (more than 14 days, right) DAD, stained for p-STING (red) and CD163 (green). Scale bars (left to right): 20 μm, 10 μm, 50 μm, 10 μm. **j**, Quantification of p-STING^+^CD163^+^ macrophages in post-mortem lungs with early and late DAD (*n* = 4). **k**, Immunohistochemistry of representative post-mortem lungs with early (left) or late (right) DAD, stained for MxA. Scale bar, 50 μm. **l**, Percentage of tissue area with MxA positivity in early and late DAD samples (*n* = 4). Data are mean ± s.d. (**b**, **c**, **e**, **h**, **j**, **l**). *P* values obtained with two-tailed Student’s t-test (**c**, **e**, **h**, **j**, **l**) and with Mann–Whitney test (**b**). Source data

**Extended Data Fig. 4:**
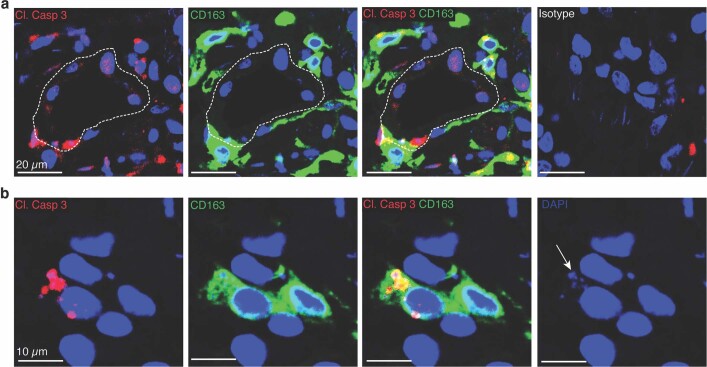
Perivascular macrophages engulf dying cells. **a**, **b**, Confocal microscopy images of representative COVID-19 skin lesion stained for CD163 (green), cleaved caspase-3 (red) and DNA (DAPI). Images are representative of 10 patients with COVID-19. Blood vessel, dashed line.

We therefore focused on the possibility that signals derived from dying (endothelial) cells promote the production of type I IFNs by macrophages. Consistent with the engulfment of dying endothelial cells, immunostaining revealed that cleaved caspase-3 fragments accumulated inside macrophages, especially in cells adjacent to the vasculature (Extended Data Fig. 4). In addition, we observed intracellular DNA foci accumulating inside IFNβ–producing macrophages (Fig. 2a, b, Extended Data Fig. 4b). On the basis of these findings, we considered that engagement of the cGAS–STING pathway, a pivotal cytosolic DNA sensing mechanism, triggers the activation of macrophages^18^. After binding DNA, cGAS synthesizes the second messenger cyclic GMP-AMP (cGAMP), which activates STING to induce cytokine responses, including type I IFNs^18^. To directly address the involvement of cGAS, we measured levels of cGAMP in whole skin extracts. Samples from patients with COVID-19, but not those from healthy donors, showed increased levels of cGAMP (Fig. 2c). Consistent with the activation of cGAS–STING signalling, phosphorylated STING (p-STING)—a selective marker of activated STING^23^—was observed in perivascular macrophages in COVID-19 lesions, but not in healthy controls (Fig. 2d, e). In addition, STING was phosphorylated in endothelial cells (Fig. 2f, g), which also contribute to type I IFN production (see above). Finally, we cultured COVID-19 skin explants overnight in the presence or absence of a small-molecule STING inhibitor, H-151 (ref. ^24^). Compared to healthy skin, COVID-19 explants exhibited significant expression of ISGs (*IFI35*, *IRF7* and *MX1*), and this response was strongly reduced by H-151 (Fig. 2h). Thus, the cGAS–STING pathway is a crucial driver of type I IFN responses in COVID-19 skin lesions.

**Extended Data Fig. 5:**
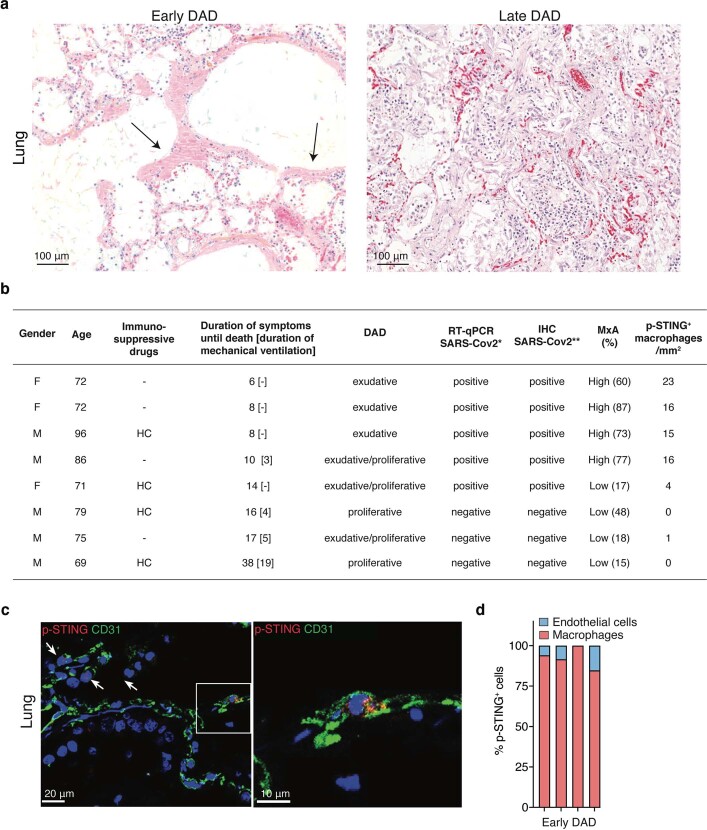
Patient characteristics and histopathological analyses of a post-mortem COVID-19-affected lung. **a**, Representative histopathology image of a COVID-19 lung in the early phase of DAD with extensive hyaline membranes (left) or in the late phase of DAD with fibrosis obliterating the alveolar lumina (right) (H&E stain). Arrows indicate hyaline membranes. **b**, Clinical parameters of the 8 patients with COVID-19 selected for the study. HC, hydroxychloroquine; Phase of the diffuse alveolar damage defined based on pure presence of hyaline membranes (exudative) or fibrotic changes (proliferative); * limit of detection is 20.8 copies per reaction (c/r) for RdRp gene, and 5.4 c/r for E gene; ** spike and nucleocapsid antibody; MxA-staining defined as high (>50% cells with intermediate to strong positive staining), or low (< 50%). **c**, Confocal microscopy images of representative COVID-19 lung section stained for CD31 (green) and p-STING (red). Arrow indicates an endothelial cell with activated STING. **d**, Proportions of CD163^+^ macrophages and CD31^+^ endothelial cells among p-STING^+^ cells in COVID-19 lungs (*n* = 4). Source data

We next sought to determine whether cGAS–STING activation also occurs in severely damaged lungs of patients with COVID-19 by post-mortem analysis^25^ (Extended Data Fig. 5a, b). We observed p-STING in macrophages and endothelial cells in some—but not all—of the lung autopsies that were analysed (Fig. 2i, j, Extended Data Fig. 5c, d). Further histopathological examination showed that lung samples that exhibited p-STING expression belonged to patients with a rapidly lethal disease course (death at fewer than 10 days after disease onset), and were characterized by signs of early diffuse alveolar damage (DAD) with extensive hyaline membrane formation (Fig. 2i, j, Extended Data Fig. 5a, b). By contrast, samples that lacked p-STING were from patients with a protracted disease course (death at more than 14 days after disease onset) and exhibited fibrotic changes that are characteristic of later phases of DAD^25^ (Fig. 2i, j, Extended Data Fig. 5a, b). In addition, samples with hallmarks for early DAD, but not late DAD, showed a type I IFN signature, as indicated by increased expression of MxA (Fig. 2k, l). Together, these analyses link SARS-CoV-2-induced tissue damage in the lung to the activation of the cGAS–STING pathway and type I IFN signalling.

## Endothelial STING response to infection

**Fig. 3:**
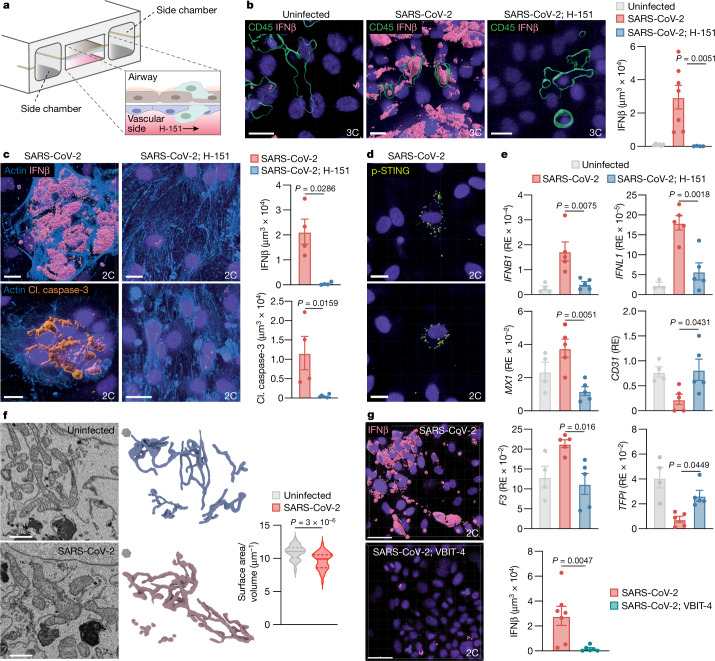
STING-dependent type I IFN production and cell death after SARS-CoV-2 infection in endothelial cells. **a**, Schematic of the three-cell component LoC model. **b**, **c**, Representative 3D images of the vascular face of uninfected or SARS-CoV-2-infected LoCs with or without vascular H-151 perfusion. ‘3C’, three-cell component (epithelial cells, endothelial cells and macrophages) in **b**; ‘2C’, two-cell component (epithelial cells and endothelial cells) in **c**. CD45^+^ macrophage (green), IFNβ (bright pink), cleaved caspase-3 (amber), actin (azure) and nuclear (purple) stainings are shown. Scale bars, 20 μm. **d**, Representative 3D images of p-STING^+^ endothelial cells (yellow). Scale bars, 20 μm. **e**, Expression levels of the indicated genes in uninfected (*n* = 4), infected (*n* = 5) and H-151-treated (*n* = 5) LoCs. *CD31* is also known as *PECAM1*. RE, relative expression. **f**, Representative volumetric electron microscopy images, 3D reconstructions and quantification of the surface-area-to-volume ratio of endothelial cell mitochondria from uninfected (*n* = 45) and infected (*n* = 43) LoCs. Solid line, mean; dashed lines, quartiles. Scale bars, 1 μm (left images); grey hexagons (middle images) represent 1 μm^3^. **g**, Representative 3D images of the vascular face of infected LoCs with or without vascular VBIT-4 perfusion. Scale bars, 50 μm. Statistics for quantification: **b**, IFNβ: uninfected (*n* = 6 fields of view (FOV)), infected (*n* = 7 FOV) and H-151-treated (*n* = 4 FOV) LoCs, *n* = 2 LoCs each; **c**, IFNβ/cleaved caspase-3: infected (*n* = 4 FOV in each case) and H-151-treated (*n* = 4/*n* = 5 FOV) LoCs, *n* = 2 LoCs each for both markers; **f**, data from *n* = 4 endothelial cells each from *n* = 1 uninfected and *n* = 2 infected LoCs; **g**, IFNβ: infected (*n* = 7 FOV) and VBIT-4-treated (*n* = 6 FOV) LoCs, *n* = 2 LoCs each. Data acquired at 3 dpi; mean ± s.e.m.; *P* values calculated by one-way ANOVA followed by Tukey’s multiple comparisons tests (**b**, **c**, **e**) or two-tailed Mann–Whitney test (**f**, **g**). Source data

**Extended Data Fig. 6:**
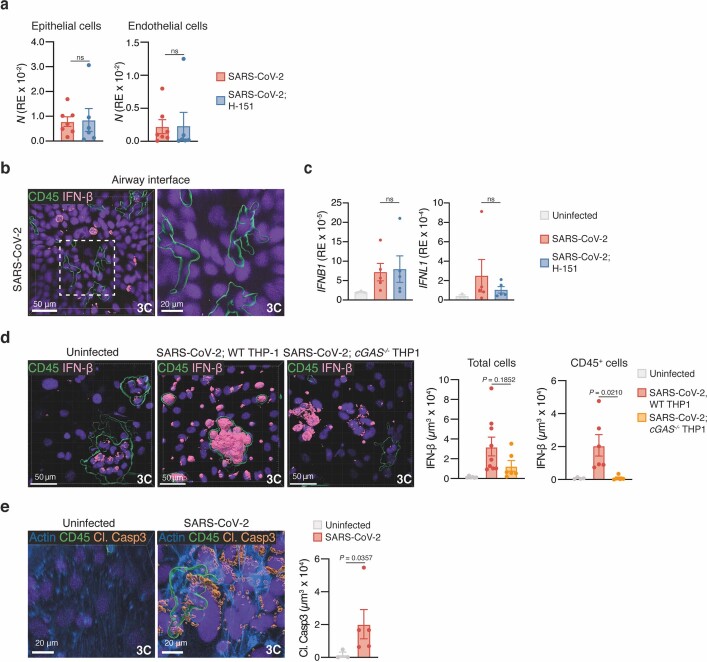
Responses from distinct cell types after SARS-CoV-2 infection in the LoC system. **a**, mRNA expression levels of SARS-CoV-2 *N* gene in epithelial and endothelial cells at 3 dpi in 2-cell component LoCs. **b**, Representative 3D views of the airway surface of a LoC infected with SARS-CoV-2 at 3 dpi. Areas with high levels of IFNβ (bright pink) are shown as surfaces. Macrophage surfaces are depicted in green, and nuclear labelling in purple. **c**, mRNA expression levels of indicated genes in the epithelial cells at 3 dpi in uninfected (*n* = 3), infected (*n* = 5), and H-151-treated (*n* = 5) 2-cell component LoCs. **d**, Representative 3D views of the vascular face from uninfected and infected LoCs; PMA-activated WT and *cGAS*^−/−^ THP-1 cells were added to vascular layer 2 days after infection. LoCs were analysed at 3 dpi by quantifying the total volume with high IFNβ expression/volume with high IFNβ expression within macrophages from uninfected (*n* = 6/ *n* = 4 fields of view), infected chips with WT THP-1 cells added (*n* = 9/ *n* = 6 fields of view) and infected chips with *cGAS*^−/−^ THP-1 cells added (*n* = 6/ *n* = 5 fields of view) across *n* = 2 LoCs in each case. **e**, Representative 3D views of the vascular face from uninfected and infected 3-component LoCs; volumes with high levels of cleaved caspase-3 (amber) are shown as surfaces. Quantification of the total volume with high cleaved caspase-3 expression from uninfected (*n* = 3 fields of view) and infected (*n* = 5 fields of view) from *n* = 1 LoC in each case. ‘3C’ refers to 3-cell component (epithelial cells, endothelial cells, and macrophages) LoCs. Bars represent mean ± s.e.m.; *P* values were calculated by a two-tailed Mann–Whitney test (**a**) or one-way ANOVA followed by Tukey’s multiple comparison tests (**c**, **d**). Source data

**Extended Data Fig. 7:**
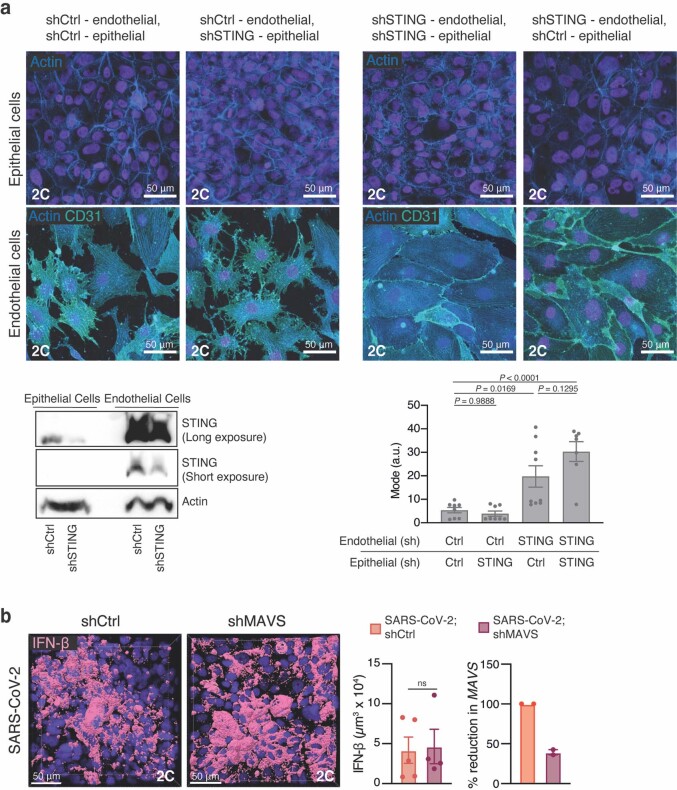
Effect of innate immune sensors on endothelial cell response after SARS-CoV-2 infection in the LoC model. **a**, Representative images of the epithelial and endothelial cells on LoC infected with SARS-CoV-2 at 3 dpi (above). Western blot characterization of STING expression in epithelial and endothelial cells treated with control (ctrl) and STING shRNAs (below, left). Modal value of CD31 expression in the endothelial layer of LoCs reconstituted with control or STING shRNA treated epithelial or endothelial cells (below, right). Each data point was calculated from a maximum intensity projection of CD31 expression from *n* = 7 (sh Ctrl - epithelial/sh STING - endothelial), *n* = *8* (sh Ctrl – epithelial/sh Ctrl – endothelial and sh STING – epithelial/sh Ctrl – endothelial) and *n* = 9 (sh STING – epithelial/sh STING = endothelial) fields of view from *n* = 1 LoC in each case. **b**, Representative 3D views at 3 dpi of the vascular surface of a LoCs reconstituted with endothelial cells treated with ctrl or MAVS shRNA and infected with SARS-CoV-2. Volumes with high levels of IFNβ (bright pink) are shown as surfaces. Quantification of the volume with high IFNβ expression from ctrl (*n* = 5 fields of view) and MAVS shRNA (*n* = 4 fields of view) from *n* = 1 LoC in each case. Reduction of MAVS mRNA in endothelial cells after shRNA transduction (right). ‘2C’ refers to 2-cell component (epithelial cells, endothelial cells) LoCs. Bars represent mean ± s.e.m.; *P* values were calculated by a one-way ANOVA test followed by Tukey multiple comparison tests (**a**) or a two-tailed Mann–Whitney test (**b**). For gel source data, see Supplementary Fig. 1. Source data

The above results suggested that, besides macrophages, endothelial cells might contribute to STING-dependent type I IFN responses in COVID-19. Although SARS-CoV-2 affects the vascular endothelium in patients, poor in vitro infection of endothelial cell cultures prevents the study of infection-associated processes in these cells^26–28^. To overcome this limitation and determine the role of STING in the endothelium, we used a lung-on-chip (LoC) model, which mimics the alveolar–capillary interface and allows for robust SARS-CoV-2-dependent activities in endothelial cells^27^ (Fig. 3a, Extended Data Fig. 6a). After infection of the alveolar epithelium, endothelial cells—but not epithelial cells—produced high levels of IFNβ, and this response was completely abolished when the STING inhibitor H-151 was perfused through the vascular channel (Fig. 3b, c, Extended Data Fig. 6b, c). Consistent with a direct engagement of STING, endothelial cells contained perinuclear foci of p-STING after infection (Fig. 3d). In addition, we verified that macrophages could contribute to the resultant type I IFN response on the vascular side in a manner that was dependent on cGAS (Fig. 3b, Extended Data Fig. 6d). Of note, we observed that the prominent virus-induced cytopathic effect in endothelial cells was also sensitive to treatment with H-151 (Fig. 3c, Extended Data Fig. 6e). By short hairpin RNA (shRNA)-mediated knockdown, we confirmed that commitment to infection-induced cell death depended on STING in endothelial cells, whereas depleting STING in the epithelial layer did not affect cell viability (Extended Data Fig. 7a). Further transcriptional analysis revealed changes of endothelial-specific activation markers (*F3*, *TFPI* and *CD31*), which were regulated by STING as H-151 effectively suppressed this response (Fig. 3e). As a control, treatment with H-151 did not affect SARS-CoV-2 transcript expression in endothelial cells (Extended Data Fig. 6a). We also tested the involvement of RNA-sensing RIG-I-like receptors and found that a knockdown of mitochondrial antiviral signalling protein (MAVS) in endothelial cells left the type I IFN response unaffected (Extended Data Fig. 7b). Together, these data reveal that STING participates in the response of endothelial cells towards SARS-CoV-2 infection by controlling distinct effector programs; namely, type I IFN signalling and endothelial cell death.

**Extended Data Fig. 8:**
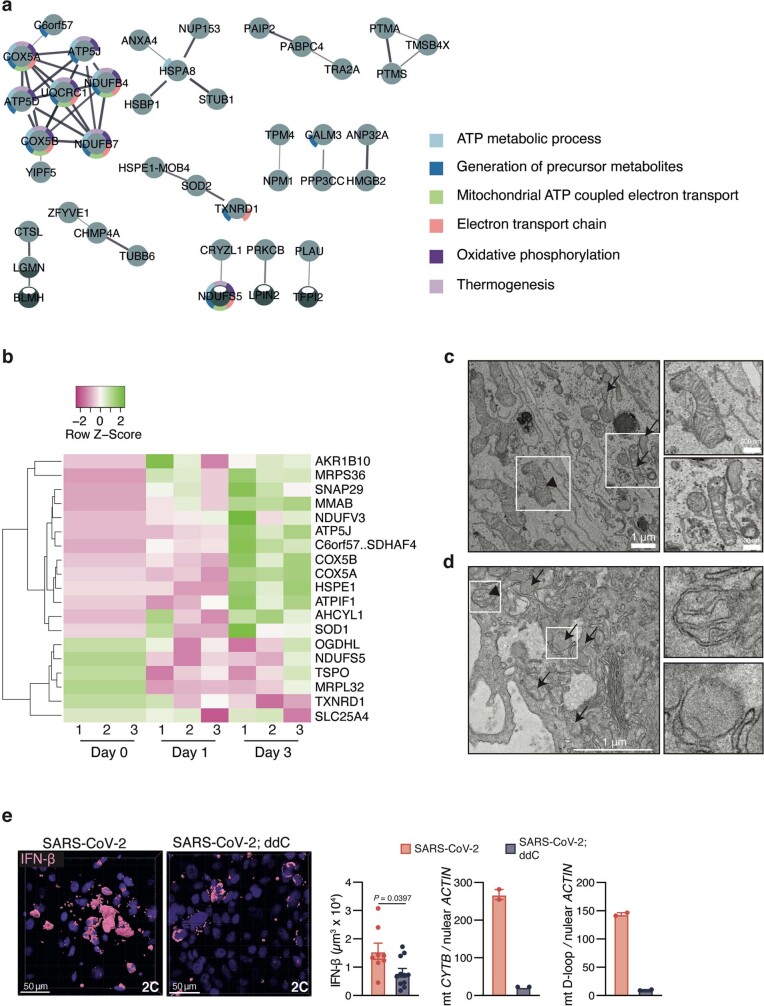
Disruption of mitochondrial homeostasis in endothelial cells after SARS-CoV-2 infection. **a**, Functional interactions between proteins with significantly altered expression identified by a pairwise analysis depicted via an interaction network generated from StringDB and clustered with the MCL algorithm. Functional annotations (GO Biological Processes/ KEGG pathways) relevant to mitochondrial function are indicated. **b**, Heat map of data from mitochondrial proteins with significantly altered expression identified via a time-course analysis. **c**, **d**, Serial block scanning electron microscope image of endothelial cells in LoC system (**c**) or transmission electron microscopy image of endothelial cells in COVID-19 skin lesion (**d**). Arrows indicate damaged mitochondria with loss of cristae morphology with a magnified inset at bottom right. As control, arrowhead indicates an intact mitochondrium with a magnified inset at top right. **e**, Representative 3D views at 3 dpi of the vascular surface of a LoCs reconstituted with endothelial cells treated or untreated with 20 μM ddC for 7 days in total and infected with SARS-CoV-2. Total mtDNA in endothelial cells was quantified by qPCR (right). Volumes with high levels of IFNβ (bright pink) are shown as surfaces. Quantification of the volume with high IFNβ expression from infected (*n* = 11 fields of view) and ddC treated infected (*n* = 8 fields of view) from *n* = 1 and *n* = 2 LoCs respectively. ‘2C’ refers to 2-cell component (epithelial cells, endothelial cells) LoCs. Bars represent mean ± s.e.m.; *P* values were calculated by a two-tailed Mann–Whitney test (**e**). Source data

We next investigated the mechanism that governs STING activation in endothelial cells. To this end, we profiled the cytosol of endothelial cells after LoC infection for changes in highly expressed proteins by mass spectrometry. Analysis of the mass spectrometry data identified differences in the abundance of 75 proteins at 3 days after infection, with a particular enrichment of mitochondrial proteins (that is, mitochondrial proteins linked to the Gene Ontology (GO) terms ‘thermogenesis’ or ‘oxidative phosphorylation’) (Extended Data Fig. 8a, Supplementary Table 3). Time-course analysis confirmed a steady increase in altered expression of proteins linked to mitochondrial metabolism (Extended Data Fig. 8b). Moreover, in volumetric ultrastructural imaging in situ, the mitochondria of endothelial cells showed disrupted cristae and appeared swollen after infection, with a pronounced reduction in the surface-area-to-volume ratio (Fig. 3f, Extended Data Fig. 8c). Notably, endothelial cells containing damaged mitochondria were also detected in skin biopsies from patients with COVID-19 (Extended Data Fig. 8d). On the basis of these findings, we hypothesized that mitochondrial DNA (mtDNA) released into the cytosol might trigger cGAS upstream of STING in endothelial cells. To test this idea, we incubated endothelial cells with 2′,3′-dideoxycytidine (ddC) to deplete mtDNA (ρ^0^ cells) (Extended Data Fig. 8e). Compared to control cells, ρ^0^ cells showed significantly less production of type I IFNs after epithelial infection with SARS-CoV-2 (Extended Data Fig. 8d). In addition, inhibition of VDAC1 oligomerization by VBIT-4, which enables the passage of mtDNA fragments into the cytosol during mitochondrial stress^29^, decreased the production of type I IFNs in endothelial cells (Fig. 3g). Therefore, extending previous findings^30,31^, SARS-CoV-2 can provoke mitochondrial dysfunction, which in endothelial cells connects to activation of the cGAS–STING pathway through the release of endogenous mtDNA.

## Targeting STING in a mouse model of COVID-19

**Fig. 4:**
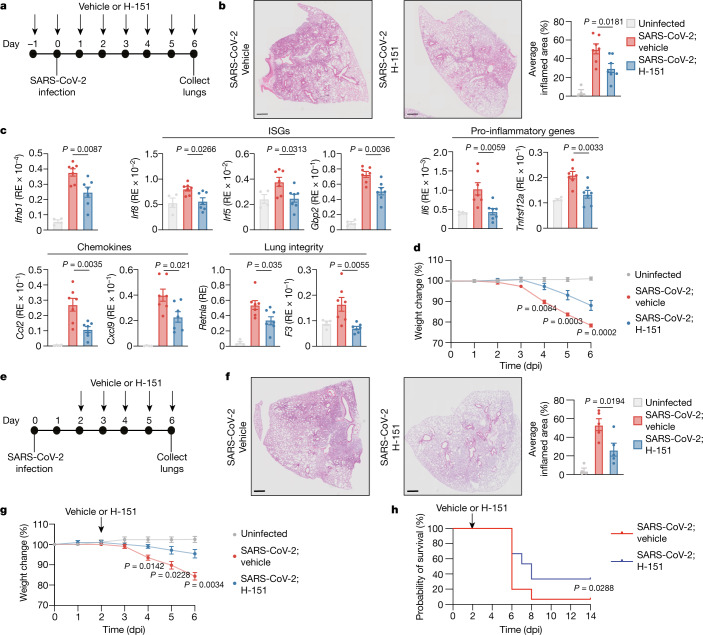
STING inhibition reduces SARS-CoV-2-induced inflammation in mice. **a**, Schematic of SARS-CoV-2 infection (intranasal; 1 × 10^4^ plaque-forming units (PFU) per mouse) and intraperitoneal administration of vehicle or H-151 (starting at 1 day before infection), related to data from **b**–**d**. **b**, Left, representative haematoxylin and eosin (H&E) images of lungs from vehicle- and H-151-treated mice. Scale bars, 500 μm. Right, average inflamed area in SARS-CoV-2 infected mice. **c**, mRNA expression levels of the indicated genes in uninfected and infected lungs at 6 dpi, analysed by RT–qPCR. **d**, Relative weight loss in mice after SARS-CoV-2 infection. **e**, Schematic of SARS-CoV-2 infection (intranasal; 1 × 10^4^ PFU per mouse) and intraperitoneal administration of vehicle or H-151 (starting at 2 dpi), related to data from **f**–**h**. **f**, Left, representative H&E images of lungs from vehicle- and H-151-treated mice. Scale bars, 500 μm, Right, average inflamed area in SARS-CoV-2 infected mice. **g**, **h**, Relative weight loss (**g**) and survival (**h**) in mice after SARS-CoV-2 infection with post-infection regimen. Numbers are: **a**–**c**, uninfected (*n* = 4), vehicle and H-151 (*n* = 7); **d**, uninfected (*n* = 8), vehicle and H-151 (*n* = 12); **e**–**g**, uninfected, vehicle and H-151 (*n* = 5); **h**, vehicle and H-151 (*n* = 15). Throughout the figure, data are mean ± s.e.m.; *P* values calculated by one-way ANOVA followed by Tukey multiple comparison tests (**b**, **c**, **d**, **f**, **g**), or by Mantel–Cox survival analysis (**h**). Mice infected with SARS-CoV-2 were age-matched (12–16 weeks) female K18-hACE2 mice. Source data

**Extended Data Fig. 9:**
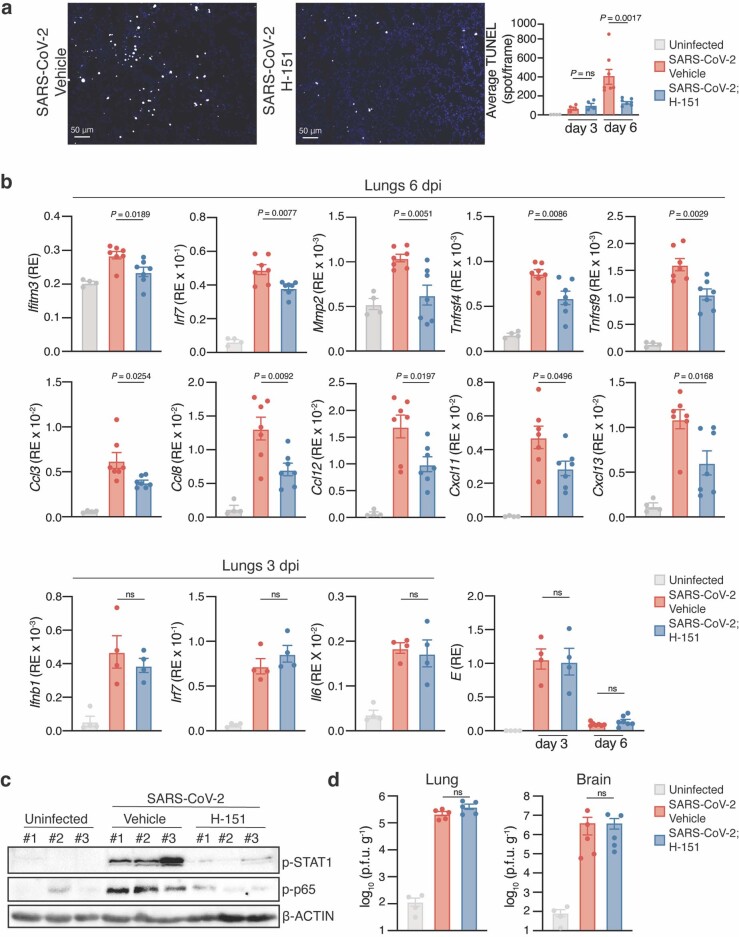
Prophylactic STING inhibition reduces pathology and inflammatory gene expression in the late stages of SARS-CoV-2 infection. **a**–**d**, Mice were infected with SARS-CoV-2 infection (intranasal; 1x10^4^ PFU/mouse) and intraperitoneal administration of vehicle or H-151 was started at 1 day prior to infection. TUNEL assay performed on the infected lung sections collected at 3 or 6 dpi is shown (**a**). mRNA levels of indicated genes isolated from uninfected and infected mouse lungs at 6 dpi were analysed by RT–qPCR (**b**). Tissue lysates from the lungs were subjected to Western blotting (**c**). Viral burden in the lungs and brains was analysed at 6 dpi by plaque assay for infectious virus (**d**). Numbers are for **a** and **b** uninfected (*n* = 4), day 3 samples (*n* = 4), day 6 samples (*n* = 7); for **d**, uninfected (*n* = 4) and infected (*n* = 5). Throughout the figure, bars represent mean ± s.e.m.; *P* values were calculated by one-way ANOVA followed by Tukey multiple comparison tests. For gel source data, see Supplementary Fig. 1. Source data

To investigate the role of STING during SARS-CoV-2 infection in vivo, we used K18-hACE2 transgenic mice, which are highly susceptible to SARS-CoV-2 infection and recapitulate important immunological features of severe COVID-19 in humans^32–36^. K18-hACE2 mice received one daily dose of H-151 starting at 16 h before SARS-CoV-2 infection and were euthanized at 3 days or 6 days post infection (dpi) (Fig. 4a). Histological examination of the lungs showed a significant reduction of inflammatory cell infiltration in H-151-treated compared to vehicle-treated mice at 6 dpi (Fig. 4b). As an independent measure of tissue pathology, we observed a prominent accumulation of dying cells (Extended Data Fig. 9a). Cell death was efficiently blocked by treatment with H-151 at 6 dpi, but not at 3 dpi (Extended Data Fig. 9a). These findings show that STING is a critical contributor to SARS-CoV-2-induced lung pathology.

When examining cytokine responses in the lungs after infection, we found that treatment with H-151 considerably decreased the expression of *Ifnb1* and ISGs (for example, *Gbp2*, *Irf5* and *Irf8*) at 6 dpi^32,35^ (Fig. 4c, Extended Data Fig. 9b). Levels of pro-inflammatory genes (*Il6* and *Tnfrsf12a*), chemokines (*Ccl2*, *Ccl3*, *Ccl12* and *Cxcl9*) and markers of lung injury (*F3* and *Retnla*) were also significantly lower in H-151-treated compared to vehicle-treated mice^35^ (Fig. 4c, Extended Data Fig. 9b). Furthermore, lung homogenates collected at 6 dpi showed strongly reduced activity of NF-κB and type I IFN signalling, as shown by decreased levels of p-p65 and p-STAT1, respectively (Extended Data Fig. 9c). Viral replication was similar in the presence or absence of H-151 at each time point, ruling out a significant effect of STING inhibition on viral replication (Extended Data Fig. 9d). Notably, at 3 dpi there was no appreciable difference in cytokine levels in the lungs between the two treatment groups (Extended Data Fig. 9b). Collectively, these data show that STING has a unique and critical function in eliciting type I IFN and inflammatory responses in the late phase of infection, which coincides with excessive tissue damage, but not with the peak of viral replication^32^. We also monitored body weight changes of infected mice over time. Compared to treatment with vehicle, treatment with H-151 significantly attenuated weight loss after infection, showing that STING has an essential role in the progression to severe disease (Fig. 4d).

**Extended Data Fig. 10:**
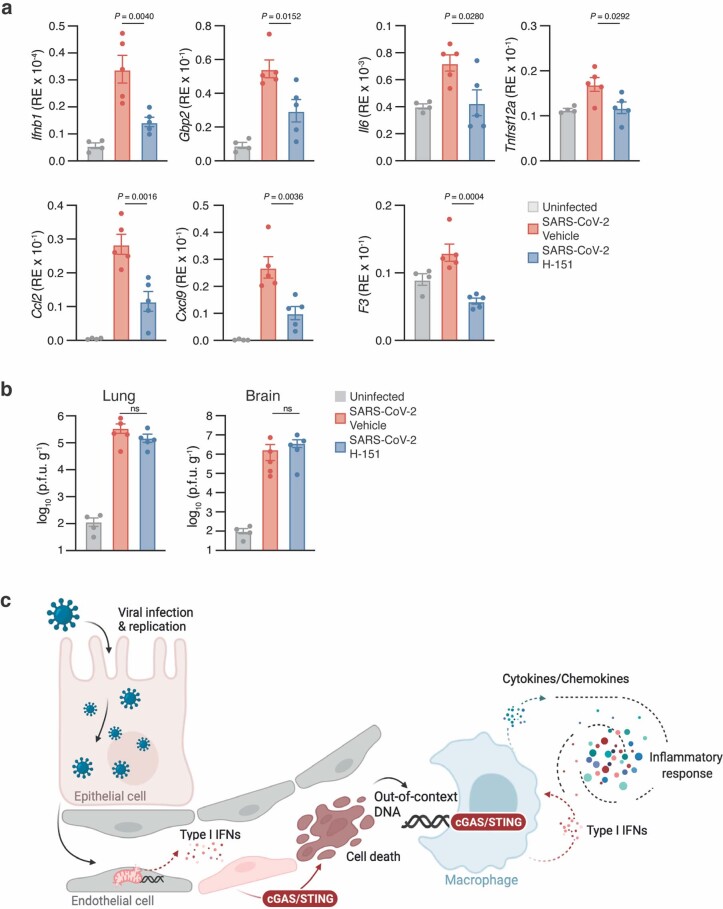
Therapeutic inhibition of STING reduces pathology and inflammatory gene expression after SARS-CoV-2 infection. **a**, **b**, Mice were infected with SARS-CoV-2 infection (intranasal; 1 × 10^4^ PFU per mouse) and intraperitoneal administration of vehicle or H-151 was started at 2 dpi. mRNA was isolated from uninfected and infected mouse lungs and relative expression of indicated genes were analysed by RT–qPCR (**a**). Viral burden in the lungs and brains was analysed at 6 dpi by plaque assay for infectious virus (**b**). **c**, Model of the involvement of the cGAS–STING pathway in severe SARS-CoV-2 infection created with biorender.com. Numbers are uninfected (*n* = 4), infected (*n* = 5) (**a**, **b**). Throughout the figure, bars represent mean ± s.e.m.; *P* values were calculated by one-way ANOVA followed by Tukey multiple comparison tests. Source data

Finally, we determined the effect of H-151 as a therapeutic agent in ongoing disease when viral loads are maximal^32^ (Fig. 4e). Mice that received H-151 at 2 dpi showed reduced pathology and decreased levels of type I IFNs and other cytokines in the lungs compared to those that were treated with vehicle only, whereas viral loads were similar between the two groups (Fig. 4f, Extended Data Fig. 10a, b). Therapeutic administration of H-151 also protected mice from weight loss and death after infection with SARS-CoV-2 (Fig. 4g, h). Together with the data above, these results corroborate the select role of STING in promoting detrimental inflammation during the late (or later) stages of the infection and highlight the therapeutic efficacy of STING inhibition, whether in a prophylactic or a therapeutic setting.

## Discussion

We have identified a central mechanism of innate immunopathology in COVID-19. Our study shows that engagement of the cGAS–STING pathway regulates two distinctive pathological features that are critically involved in the progression and severity of COVID-19—namely, endothelial dysfunction and type I IFN production (Extended Data Fig. 10c). Moreover, we establish endothelial cells and macrophages as cells at the root of maladapted cGAS–STING responses, which are driven in each cell type by a distinct underlying process. In a cell-intrinsic mode of activation, cGAS within endothelial cells is stimulated by the loss of mitochondrial homeostasis and associated accumulation of mtDNA to direct the expression of type I IFNs, activation of endothelial cells and, ultimately, cell death. By contrast, macrophage-dependent cGAS responses are more focused on type I IFN induction and result from the recognition of DNA from engulfed dying (endothelial) cells. Our finding that STING regulates endothelial cell death accords well with previous reports of endotheliopathy and vascular damage due to gain-of-function mutations in STING or after administration of highly potent STING agonists^37,38^. Consistent with previous reports in patients with COVID-19 (ref.^26^), endothelial cells in our LoC studies contain viral elements, arguing for direct viral involvement in triggering mitochondrial dysfunction and, in turn, the activity of the cGAS–STING pathway. Along these lines, cell-autonomous activation of cGAS–STING signalling has recently been suggested to contribute to the NF-κB-dependent production of cytokines in SARS-CoV-2-infected human epithelial cell lines^39^, which indicates that the pathway could have a more extensive role in COVID-19-associated cytokine responses. Given the importance of vascular damage in COVID-19, establishing the precise upstream cause (or causes) of endothelial cell involvement in viral activities is an area that warrants future investigation^21,40^.

Many reports have pointed to the context-dependent roles of type I IFNs during infection with highly pathogenic coronaviruses^3,5,6,9–11,17,41,42^. Our study adds to these reports by indicating that the signalling mechanisms that underlie the induction of beneficial (early) versus detrimental (delayed) type I IFN responses are distinct. In a direct mode of recognition, rapid detection of viral RNA by Toll-like receptors 3 and 7 and RIG-I-like receptors initiates a type I IFN response that confers antiviral protection to the host^43–46,47^. By contrast, activation of the cGAS–STING pathway by DNA emerges from a collateral host response to tissue damage. This explains the involvement of the pathway in type I IFN production at the later stages of infection, eventually sustaining deleterious inflammation. Notably, STING-dependent induction of type I IFNs is compromised in bats, which raises the possibility that this immunological adaption could account for the increased tolerance of these animals to highly pathogenic coronavirus infection^48^.

In summary, our study has implications both for the understanding of how the innate immune system contributes to detrimental outcomes of SARS-CoV-2 infection and for current efforts to define new therapeutic paradigms for more efficient treatment modalities in COVID-19.

## Methods

### Patient data and samples

Studies were approved by the institutional review and privacy boards of the Lausanne University Hospital CHUV, and the local ethics committee, in accordance with the Helsinki Declaration: CER-VD 2020-02204 for studies using skin samples, and CER-VD 2020-01257 for studies using post-mortem lungs. For COVID-19 skin samples, 10 consecutive patients presenting with moderate-to-severe COVID-19 disease and associated skin manifestations, hospitalized at CHUV from the beginning of the COVID pandemic in March 2020 were selected. All patients had positive PCR tests from nasal swab for SARS-CoV-2 and a clinical diagnosis of COVID-19. Comprehensive information on comorbidities, immunosuppressive treatment, type of skin manifestation and time from first symptoms are given in Extended Data Fig. 1b. In addition, the maximal disease severity score, determined according to the NIH ordinal scale and Sequential Organ Failure Assessment (SOFA) is provided. The score is defined as (1) not hospitalized with no limitation of activities; (2) not hospitalized with limitation of activities and/or home oxygen requirement; (3) hospitalized but not requiring supplemental oxygen and no longer requiring ongoing medical care; (4) hospitalized and not requiring supplemental oxygen but requiring ongoing medical care; (5) hospitalized requiring supplemental oxygen; (6) hospitalized requiring non-invasive ventilation or the use of high-flow oxygen devices; (7) hospitalized receiving invasive mechanical ventilation or extracorporeal membrane oxygenation; and (8) death. Control skin samples included skin lesions from CLE (*n* = 11), plaque-type psoriasis (*n* = 21), atopic dermatitis (*n* = 16), lichen planus (*n* = 5) and healthy skin (*n* = 4). All patient biopsies were taken after informed consent was obtained and clinical diagnosis of the disease was histologically confirmed.

For the lung studies, eight post-mortem examinations of patients who died from COVID-19 at CHUV since March 2020 were included. All patients had positive PCR-tests from nasal swabs for SARS-CoV-2 and a clinical diagnosis of COVID-19. Patient information including comorbidities, immunosuppressive treatment, type of DAD, duration of symptoms until death and days of mechanical ventilation are given in Extended Data Fig. 6 and in Berezowska et al.^25^.

### Assessment of the endotheliopathy index in skin lesions

For scoring the endotheliopathy in skin samples, the outer and inner diameters of the post-capillary vessels of the superficial and middle dermis were measured and the endothelial swelling index was calculated as the ratio of the two diameters.

### Mice

Twelve-to-sixteen-week-old female K18-hACE C57BL/6J transgenic mice (strain: 2B6.Cg-Tg(K18-ACE2)2Prlmn/J) were obtained from The Jackson Laboratory. Mice were housed in groups and fed standard chow diets. Mice were housed in groups of up to five mice per cage at 18 °C–24 °C ambient temperature with 40–60% humidity. Mice were maintained on a 12-h light–dark cycle from 06:00 to 18:00. Food and water were available ad libitum. Mice were administered 1 × 10^4^ PFU of SARS-CoV-2 by intranasal administration. Virus inoculations were performed under anaesthesia that was induced and maintained with ketamine hydrochloride and midazolam, and all efforts were made to minimize animal suffering. The cages were assigned randomly for vehicle and H-151 treatment groups after the SARS-CoV-2 infection, and the experimenter was not blinded afterwards. Mice intraperitoneally received daily either dimethyl sulfoxide (DMSO) as vehicle or 750 nmol H-151 in 200 µl PBS 5% Tween-80. Mice were euthanized on the indicated day and immediately dissected for transcardial perfusion with 20 ml ice-cold PBS. Lungs and brains were collected. Half of each lung lobe was fixed in 4% PFA for histological analysis, and the other half of the lobes was chopped and stored for further analysis. For the survival study, mice were administered SARS-CoV-2 by intranasal administration as described above. Mice were euthanized when they reach one of the humane end point criteria: (1) more than 25% weight loss; (2) paralysis; (3) severe dyspnea. Animal experiments were approved by the Service de la Consommation et des Affaires Vétérinaires of the canton of Vaud (Switzerland) and were performed in accordance with the respective legal regulations.

### Plaque-forming assay

Lung and brain of the mice were homogenized in Vero-E6 cell-culture medium (DMEM + 10% FBS + P/S). Homogenized mix was centrifuged at 400*g* for 10 min. The supernatant was analysed for the viral content. Vero-E6 cells were seeded in a 12-well plate at a density of 2.5 × 10^5^ cells per well. Cells were washed with PBS and inoculated with viruses serially diluted in cell-culture medium. One hour after the infection, cells were washed with PBS, and overlaid with 0.8% Avicel (GP 3515) mixed at 1:1 with DMEM supplemented with 4% fetal bovine serum, 200 units ml^−1^ penicillin and 200 μg ml^−1^ streptomycin. After 72 h of incubation, the overlay was removed and cells were washed with PBS, fixed with 4% PFA and stained with crystal violet.

### Immunofluorescence and immunohistochemistry analysis

Formalin-fixed paraffin-embedded (FFPE) skin blocks were cut into 6-μm sections and placed on slides. Sections were first deparaffinized and rehydrated, then Heat-Induced Epitope Retrieval (HIER) was performed and sections were permeabilized with PBS 0.01% Triton. Samples were stained with primary antibodies (Supplementary Table 1) for 2 hours at room temperature. For immunofluorescence analysis, sections were then stained with fluorescently labelled secondary antibodies (Supplementary Table 1) for 30 min at room temperature. For immunohistochemistry, sections were stained with HRP-conjugated secondary antibodies followed by DAB staining and Mayer counterstaining. For RNA fluorescence in situ hybridization (FISH), *IFNB1* mRNA was detected in skin using RNAScope Multiplex Fluorescent V2 Assay following the manufacturer’s instructions (Advanced Cell Diagnostics, Inc.). Co-staining of sections with mouse anti-human CD163 (Diagnostic Bio Systems) was performed as described above. Images were acquired with a Zeiss LSM 700 confocal microscope and analysed with Zen 2010 software. For cell quantification, slides were digitalized using the PANNORAMIC 250 Flash digital scanner (3DHISTECH Ltd) and cell types were quantified using the QuantCenter plug-in 2.2 of Caseviewer 2.4 software.

For LoC samples, the fixed LoCs were permeabilized with 0.1% Triton, 2% saponin and incubated with a blocking solution of 2% bovine serum albumin (BSA) for 1 h followed by overnight incubation with the primary antibody (1:100 dilution) in the blocking buffer at 4 °C. The chip was then incubated with secondary antibodies (1:300 dilution) for 1 h at room temperature. A list of the primary antibodies and concentrations used is included in Supplementary Table 1. F-actin was stained using Sir-Actin dye in the far-red (Spirochrome) at 1 µM for 30 min concurrently with Hoechst staining.

Mouse lungs were cut into 3-μm sections. The extent of lung inflammation was quantified as the average percentage of lung surface area in which the alveolar wall is thickened with at least 50% decreased airspace area and was assessed by two independent investigators using three lung sections per mouse. TUNEL staining was performed using a commercially available kit (Promega) according to the manufacturer’s instructions. Imaging was performed using the Zeiss Axioplan fluorescence microscope with the use of Axiovision software. Three fields were selected randomly from each lung piece. TUNEL-positive cells were quantified by automated counting performed by image analysis software (ImageJ).

### RNA extraction

Excised 4-mm skin biopsies were immediately snap-frozen in liquid nitrogen and stored at −80 °C until processing. RNA was isolated using the TRIzol/chloroform method and a tissue homogenizer (Thermo Fisher Scientific). All isolated RNA had an A260/A280 value ≥1.7 and RNA integrity was analysed on a Fragment analyser (Agilent). Mouse lung pieces were lysed in TRIzol (Thermo Fisher Scientific) and RNA was isolated according to the manufacturer’s instructions. RNA from cells in lung-on-chip experiments was isolated by using the RNeasy Micro Kit (Qiagen) according to the manufacturer’s instructions.

### NanoString analysis

mRNA expression of 600 targets was analysed with the nCounter Human Immunology V2 panel including 20 customized probes (Nanostring Technologies, Seattle, WA, USA) on the nCounter platform (Nanostring Technologies) using 100 ng of RNA per skin sample. This commercial panel was extensively validated in-house for accuracy, repeatability and reproducibility before analysing the study samples. A quality check was run for each sample before including it into the analysis. Data were normalized and analysed using either nSolver 4.0 (Nanostring Technologies) or ROSALIND (ROSALIND Inc., San Diego, CA). Housekeeping probes to be used for normalization are selected based on the geNorm algorithm as implemented in the NormqPCR R library^49^. Clustering of genes for the final heatmap of differentially expressed genes was done using the PAM (Partitioning Around Medoids) method using the fpc R library (https://cran.r-project.org/web/packages/fpc/index.html) that takes into consideration the direction and type of all signals on a pathway and the position, role and type of every gene. The *z*-scores of each gene were then calculated for the selected patients to generate heatmaps and determine specific classifiers.

### STING inhibition in skin explants

Six-millimetre skin biopsies from healthy individuals or patients with COVID-19 were cut into three equal pieces and one piece was snap-frozen to measure the baseline gene expression. The two remaining pieces were cultured in 200 μl of DMEM 10% FBS, 1% penicillin–streptomycin in the presence or not of 0.5 μg ml^−1^ of H-151 for 15 h. Skin biopsies were then homogenized in TRIzol to perform RNA extraction followed by NanoString analysis as described above.

### 2'3'-cGAMP enzyme-linked immunosorbent assay

Six-millimetre skin punch biopsies were lysed in Pierce RIPA Buffer using a tissue homogenizer (Thermo Fisher Scientific). Protease inhibitor cocktail (Sigma) was added to prevent protein degradation. Thirty micrograms of the lysate was used to measure 2'3'-cGAMP concentrations by enzyme-linked immunosorbent assay (ELISA) and according to the manufacturer’s instructions (Cayman Chemical).

### Ultrastructural analysis of the skin

For transmission electron microscopy, the skin biopsies were fixed in 2% glutaraldehyde in 0.1M sodium cacodylate buffer, pH 7.4. Samples were then post-fixed in 1% OsO_4_/1.5% potassium ferrocyanide in aqua bidest for 2 h, block stained with uranyl acetate (2% in distilled water), dehydrated in alcohol (stepwise 50–100%), immersed in propylenoxide and embedded in glycidyl ether (polymerized 48 h at 60 °C; SERVA, Electrophoresis GmbH, Heidelberg, Germany). Semi-thin and ultra-thin sections were cut with an ultramicrotome (Ultracut, Reichert Inc., Vienna, Austria). Ultra-thin sections (30 nm) were mounted on copper grids and analysed on a Zeiss LIBRA 120 transmission electron microscope (Carl Zeiss, Oberkochen, Germany) operating at 120 kV.

### RT–qPCR analysis

For mouse lung samples, RNA was reverse-transcribed using the RevertAid First Strand cDNA Synthesis reagents (Thermo Fisher Scientific), and quantitative PCR with reverse transcription (RT–qPCR) was performed in duplicate using Maxima SYBR Green Master Mix (Thermo Fisher Scientific) on QuantStudio 6/7 qPCR instruments. For LoC samples, RNA was reverse-transcribed using the SuperScript IV First-Strand Synthesis System with random hexamers (Invitrogen), and RT–qPCR reactions were prepared with SYBR Green PCR Master Mix (Applied Biosystems) on the ABI PRISM 7900HT System (Applied Biosystems). Amplicon specificity was confirmed by melting-curve analysis. The primer sequences are listed in Supplementary Table 2.

### Immunoblotting

SDS-loading buffer was mixed with the lung lysates in RIPA buffer and denatured at 95 °C for 10 min. Lysates were separated by 10% SDS–PAGE and transferred onto nitrocellulose membranes. Blots were incubated with anti-p-p65 (Ser468) (1:1,000 dilution), and anti-p-STAT1 (Tyr 701) (1:1,000 dilution) (Cell Signaling) and anti-β-actin-HRP (1:2,000 dilution) (Santa Cruz Biotechnology). Proteins were visualized with the enhanced chemiluminescence substrate ECL (Pierce, Thermo Fisher Scientific) and imaged using the ChemiDoc XRS Biorad Imager and Image Lab Software 5.1. Uncropped images are presented in Supplementary Fig. 1.

### Primary human cell culture and culture of macrophage cell lines

Primary human alveolar epithelial cells (epithelial cells) and human lung microvascular endothelial cells (endothelial cells) were obtained from a commercial supplier (Cell Biologics). All chips were reconstituted with epithelial cells seeded directly on the LoC without any additional in vitro culture. Endothelial cells were passaged 3–5 times before seeding in the LoC devices. Experiments were performed with cells from at least two donors.

Peripheral blood mononuclear cells from buffy coat (Interregional Blood Transfusion SRC) were obtained from anonymized donors and isolated using a Biocol Separation procedure as per the manufacturer’s instructions. One week before seeding the macrophages in the LoC devices, a cryopreserved aliquot was cultured in a T-75 flask (TPP) in RPMI supplemented with 10% FBS. CD14^+^ monocytes were isolated using positive selection (CD14 ultrapure isolation kit, Miltenyi Biosciences), embedded in hemispherical domes of basement membrane extract (BME-2, Cultrex) in 24-well plates, and cultured in RPMI medium supplemented with 10% FBS, 20 ng ml^−1^ recombinant human macrophage-colony stimulating factor protein (M-CSF) and 100 U l^−1^ of penicillin–streptomycin solution (Thermo Fisher Scientific). The monocytes were differentiated for 7 days. On the day of seeding into the LoC, the BME domes were first disrupted by scraping with a P1000 pipette. The mechanically dissociated hydrogel was then brought to semi-liquid state by adding 500 µl of ice cold RPMI medium per well. The BME-RPMI suspension was then centrifuged at 200*g* for 5 min in a 15-ml Falcon tube pre-coated with 1% BSA in PBS and resuspended in 4–5 ml of ice-cold Cell Recovery Solution (Corning) over ice for 20–30 min. Occasionally, the solution was sheared with a fire-polished glass pipette to ensure the complete removal of the macrophages from the BME hydrogel. The cell suspension was then washed twice with 10 ml of RPMI medium/10% FBS to remove remaining traces of the cell recovery reagent. If required, BME-2 suspension was sometimes incubated in 2–3 ml trypsin to remove remaining fragments of BME-2, and the washing step was repeated again. Isolated macrophages were resuspended in epithelial cell medium and passed through a 40-μm filter to obtain a single-cell suspension of macrophages.

Wild-type and *cGAS*^−/−^ THP-1 cells were cultured according to the suppliers’ instructions. THP-1 cells were differentiated with 5 ng ml^−1^ PMA for 3 days and transferred to the vascular channel of the LoC at 2 dpi.

### Production of lentiviral vectors and transduction of primary epithelial and endothelial cells

HEK-293T cells were a gift from the laboratory of D. Trono. HEK-293T cells were transfected with pCMVDR8.74, pMD2.G plasmids and the puromycin-selectable pLKO.1-puro lentiviral vector containing the shRNA for human STING (5′-CATGGTCATATTACATCGGAT-3′) and human MAVS (5′-CAAGTTGCCAACTAGCTCAAA-3′) by the calcium phosphate precipitation method. The supernatant containing lentiviral particles was collected at 48 and 72 h, pooled and concentrated by ultracentrifugation. Primary endothelial cells (shRNA for STING and MAVS) and primary alveolar epithelial cells at passage 5 (shRNA for STING only) were transduced with the lentiviral vectors by directly adding 10 μl to the culture medium; transduced cells were selected by adding 1 μg ml^−1^ puromycin to the medium 48 h after the transduction.

### Generation of SARS-CoV-2 stocks

VeroE6 cells and a clinical isolate of SARS-CoV-2 were a gift from the laboratory of C. Tapparel. SARS-CoV2/Switzerland/GE9586/2020 was isolated from a clinical specimen in the University Hospital in Geneva in Vero-E6 cells. Vero-E6 cells were infected and supernatant was collected at 3 dpi, clarified, aliquoted and frozen at −80 °C and subsequently titrated by plaque assay in Vero-E6. Viruses used for the LoC and animal experiments in this manuscript were at passage 2 and passage 4, respectively, in Vero-E6 cells.

### Infection of the LoC model with SARS-CoV-2

LoC devices were purchased from a commercial vendor (Emulate). For a small subset of experiments for RT–qPCR measurements in uninfected controls, devices fabricated in-house with similar dimensions (but without a stretching channel) using porous membranes supplied by a commercial vendor were used (BiOND). A detailed protocol for the establishment of the LoC model has been described previously^24^. In brief, devices were coated with ECM solution of 150 µg ml^−1^ bovine collagen type I (AteloCell, Japan) and 30 µg ml^−1^ fibronectin from human plasma (Sigma-Aldrich). For the three-component model with primary macrophages, differentiated primary human macrophages were seeded directly on the PDMS membrane 1–2 h before seeding of the endothelial cells on the basolateral side of the membrane and epithelial cells on the apical side. For experiments with ρ^0^ endothelial cells, the endothelial cells were incubated with ddC for 3–5 days before infection. The chip was incubated overnight with complete epithelial and endothelial medium in the respective channels under static conditions. Thereafter, a reduced medium for the air–liquid interface (ALI) was flowed through the vascular channel and the epithelial face was incubated with epithelial base medium supplemented with 1 µM dexamethasone (Sigma-Aldrich). This medium was replaced daily for the following 2–3 days. Thereafter, the chips were maintained overnight at an ALI and then then transferred to the biosafety level 3 (BSL-3) facility for SARS-CoV-2 infection. Here, an aliquot of virus-containing supernatant was diluted approximately 20-fold in epithelial cell medium without FBS to generate the inoculum that corresponded to an infectious dose of 400–600 PFU in a volume of 30 µl. This volume was then added to the apical channel of each LoC, and the LoC was incubated for an hour at 37 °C and 5% CO_2_. Thereafter, the LoC was returned to ALI. For LoCs treated with the STING or the VDAC oligomerization inhibitor, H-151 (1 µM) or VBIT-4 (1 µM) was perfused through the vascular channel after infection and maintained over the course of 3 days. Infection was terminated at specified time points and the LoCs processed for RNA extraction or by fixation with freshly prepared 4% paraformaldehyde for a period of 30 min.

### Confocal imaging and image analysis of LoCs

Infected and control LoCs were imaged using a Leica SP8 confocal microscope with a white light laser. LoCs were imaged with a 25× water immersion objective (NA = 0.95, Leica), with standard settings (voxel size 227.27 × 227.27 × 300 nm^3^) across chips labelled the same way. *Z*-stacks were subsequently deconvolved using the Huygens Deconvolution Software (Scientific Volume Imaging) and 3D views were rendered using Imaris (Bitplane). Maximum intensity projects were rendered using ImageJ. The following parameters were used for generation of the surfaces in Imaris for the visualization of IFNβ, cleaved caspase-3, macrophages and p-STING. In each case, uninfected control chips and/or infected chips and/or treated chips from the same experiment were immunostained and imaged together, to control for differences in the immunofluorescence intensities across antibody aliquots, imaging conditions, and microscopes. Chips from the same experiment were analysed using the same Imaris parameters. Three-cell component chips in Fig. 3b, Extended Data Fig. 6b, IFNβ: manual threshold: 110, smoothing: 0.455 µm. Three-cell component chips in Extended Data Fig. 6d, IFNβ: manual threshold: 110, smoothing: 0.455 µm. Two-cell component chips in Fig. 3c, Extended Data Fig. 7b, IFNβ: manual threshold: 45, smoothing: 0.455 µm. Three-cell component chips in Extended Data Fig. 6e, cleaved caspase-3: manual threshold: 110, smoothing: 0.8 µm. Two-cell component chips in Fig. 3c, cleaved caspase-3: manual threshold: 110, smoothing: 0.8 µm. Two-cell component chips in Fig. 3d., p-STING: manual threshold: 110, smoothing: 0.8 µm. Three-cell component chips in Fig 3b, CD45: manual threshold: 110, smoothing: 1 µm. Two-cell component chips in Fig. 3g, Extended Data Fig. 8d, IFNβ; manual threshold: 30; smoothing: 0.455 µm.

### Sample preparation for proteomic analysis

Cells from the vascular and apical faces of the LoC devices were extracted in a sequential manner by instillation of 0.25% Trypsin-EDTA solution (Gibco) in the vascular channel followed by the apical channel. Cells were centrifuged at 300*g* for 5 min and washed twice with PBS solution to eliminate extracellular matrix components. Pelleted cells were then resuspended in a 20-µl solution of 100 mM HEPES pH 8 and 5 mM tris(2-carboxyethyl)phosphine and heat-inactivated at 95 °C for 10 min before removal from the BSL-3 facility, and stored at −20 °C for subsequent processing at the Proteomics Core Facility. Here cells were vacuum-centrifuged to near dryness and resuspended in 9 μl of 100 mM HEPES pH 8 and 10 mM tris(2-carboxyethyl)phosphine. Samples were first heated for 20 min at 95 °C with permanent shaking and then sonicated in a water bath for 15 min. Extracted proteins were alkylated with 1 μl of 400 mM chloroacetamide for 30 min at 37 °C in the dark with permanent shaking. Proteins were digested overnight using 400 ng mass spectrometry grade trypsin with permanent shaking. The resulting peptides were desalted on C18 StageTips^50^ and dried by vacuum centrifugation. Peptides were reconstituted in 8 μl HEPES 100 mM pH 8 and labelled with 3 µl of isobaric tags (TMT 20 µg μl^−1^ in pure acetonitrile) for 90 min at room temperature. The labelling reaction was stopped with addition of 50% hydroxylamine (final concentration 0.4% (v/v)) for 15 min. A small fraction of the labelled peptides was mixed in a 1:1 ratio across all samples and analysed with a single shot control liquid chromatography–tandem mass spectrometry (LC–MS/MS) run to evaluate the mixing accuracy. On the basis of the results of this control run, the remaining labelled peptides were mixed in equal amounts, vacuum-centrifuged and fractionated into eight fractions using the Pierce High pH Reversed-Phase Peptide Fractionation Kit following the manufacturer’s instructions. The eight fractions were dried by vacuum centrifugation and stored at −20 °C.

### Mass spectrometry

Peptides were resuspended in 2% acetonitrile, 0.1% FA and analysed on a Lumos Fusion Orbitrap Mass Spectrometer online connected to a Dionex Ultimate 3000 RSLC nano UPLC system. A capillary precolumn (Acclaim Pepmap C18, 3 μm 100 Å, 2 cm × 75 μm inner diameter) was used for sample trapping and cleaning. Analytical separations were performed at 250 nl min^−1^ over 150-min biphasic gradients on a 50-cm-long in-house packed capillary column (75 μm inner diameter, ReproSil-Pur C18-AQ 1.9 μm silica beads, Dr. Maisch). Acquisitions were performed through the top speed data-dependent acquisition mode using a 3 s cycle time. First MS scans were acquired at a resolution of 120,000 (at 200 *m*/*z*) and the most intense parent ions were selected and fragmented by high energy collision dissociation (HCD) with a normalized collision energy (NCE) of 37.5% using an isolation window of 0.7 *m*/*z*. Fragmented ion scans were acquired with a resolution of 50,000 (at 200 *m*/*z*) and selected ions were then excluded for the following 120s.

### Mass spectrometry data analysis

Raw data were processed using SEQUEST, Mascot, MS Amanda^51^ and MS Fragger^52^ in Proteome Discoverer v.2.4 against a concatenated database consisting of the Uniprot human reference proteome (release 2020_10) and Uniprot SARS-CoV-2 reference proteome (release 2020_10). Enzyme specificity was set to trypsin and a minimum of six amino acids was required for peptide identification. Up to two missed cleavages were allowed and a 1% false discovery rate (FDR) cut-off was applied both at peptide and at protein identification levels. For the database search, carbamidomethylation (C) and TMT tags (K and peptide N termini) were set as fixed modifications whereas oxidation (M) was considered as a variable. The resulting text files were processed through in-house written R scripts (v.3.6.3). Two steps of normalization were applied: sample loading (SL) and trimmed mean of M-values (TMM) normalization. The SL normalization^53^ assumes that total protein abundances are equal across the TMT channels; therefore, the reporter ion intensities of all spectra were summed and each channel was scaled according to this sum, so that the sum of reporter ion signals per channel equals the average of the signals across samples. Subsequently, the TMM normalization step was applied using the package EdgeR (v.3.26.8)^54^. This normalization step works on the assumption that most of the protein abundances do not change across samples therefore, it calculates normalization factors according to these presumed unchanged protein abundances. Differential protein expression analysis was performed using the R bioconductor package limma (v.3.40.6, 2020-02-29)^55^, followed by the Benjamini–Hochberg multiple-testing method^56^. *P* values lower than 0.00128 (FDR < 0.05) and absolute log_2_-transformed fold change (log_2_FC) > 0.5 were considered as significant. For the time-course study, all quantified proteins were monitored. The significant temporal dynamics were defined with the timecourse package in R Bioconductor, which uses a multivariate empirical Bayes model to rank proteins^57^. Replicate time-course data can be compared allowing for variability both within and between time points. The mb.long method was used to calculate the moderated Hotelling T^2^ statistic, specifying a one-dimensional method (method = “1D”), in which significant proteins change over the time course. The null hypothesis is that the protein temporal profile is equal to 0.

### Statistics and reproducibility

Statistical analyses are described in each figure legend. For experiments combining several groups, an ordinary one-way ANOVA test was used. Statistical significance was determined using Prism v.8.0 software (GraphPad). Significant differences between groups were determined by post-hoc Tukey’s multiple comparisons tests, unless specified otherwise, *P* > 0.05 was considered non-significant. The Student’s *t*-test or the Mann–Whitney test was used to assess the *P* value when comparing only two groups. For LoC studies, fields of view from a given LoC are considered as biological replicates, and the number of LoCs corresponds to the number of times the experiment was repeated. Images of p-STING^+^ endothelial cells in Fig. 3d are from *n* = 2 fields of view from *n* = 1 LoC. Data of mitochondria with loss of cristae morphology are representative slices from volumetric electron microscopy imaging of *n* = 4 endothelial cells from *n* = 2 infected LoCs. Data from patient samples were obtained from *n* = 3 independent experiments and quantifications for histological analysis were performed by *n* = 2 independent investigators.

### Reporting summary

Further information on research design is available in the Nature Research Reporting Summary linked to this paper.

## Online content

Any methods, additional references, Nature Research reporting summaries, source data, extended data, supplementary information, acknowledgements, peer review information; details of author contributions and competing interests; and statements of data and code availability are available at 10.1038/s41586-022-04421-w.

## Supplementary information

Supplementary Figure 1 This file contains full scans for all western blots and the in-gel fluorescence images, in Supplementary Fig. 1.

Reporting Summary

Supplementary Table 1 A list of antibodies used in this study.

Supplementary Table 2 A list of qRT–PCR primers and shRNA sequences in this study.

Supplementary Table 3 Data for pairwise comparison (limma analysis) and timecourse analysis of the proteomics data in this study.

## Data Availability

Full scans for all western blots and the in-gel fluorescence images are provided in Supplementary Fig. 1 and the limma and timecourse analyses of the proteomics data are provided in Supplementary Table 3. Raw data supporting the findings of this study have been deposited at Zenodo and are publicly available at 10.5281/zenodo.5818157. The proteomics dataset generated during this study has been deposited in the PRIDE database with accession code PXD030753. The NanoString dataset generated during this study has been deposited at the Gene Expression Omnibus (GEO) database with accession code GSE193068. Source data are provided with this paper.

## Source data

Source Data Fig. 1
Source Data Fig. 2
Source Data Fig. 3
Source Data Fig. 4
Source Data Extended Data Fig. 2
Source Data Extended Data Fig. 3
Source Data Extended Data Fig. 5
Source Data Extended Data Fig. 6
Source Data Extended Data Fig. 7
Source Data Extended Data Fig. 8
Source Data Extended Data Fig. 9
Source Data Extended Data Fig. 10

## Code availability

Custom scripts in R for the analysis of proteomics data have been deposited at Zenodo and are publicly available at 10.5281/zenodo.5818157.

## References

[1] Huang C (2020). Clinical features of patients infected with 2019 novel coronavirus in Wuhan, China. Lancet.

[2] Gupta A (2020). Extrapulmonary manifestations of COVID-19. Nat. Med..

[3] Lucas C (2020). Longitudinal analyses reveal immunological misfiring in severe COVID-19. Nature.

[4] Nienhold R (2020). Two distinct immunopathological profiles in autopsy lungs of COVID-19. Nat. Commun..

[5] Park A, Iwasaki A (2020). Type I and type III interferons—induction, signaling, evasion, and application to combat COVID-19. Cell Host Microbe.

[6] Lee JS, Shin E-C (2020). The type I interferon response in COVID-19: implications for treatment. Nat. Rev. Immunol..

[7] Hadjadj J (2020). Impaired type I interferon activity and inflammatory responses in severe COVID-19 patients. Science.

[8] Chua RL (2020). COVID-19 severity correlates with airway epithelium–immune cell interactions identified by single-cell analysis. Nat. Biotechnol..

[9] Zhou Z (2020). Heightened innate immune responses in the respiratory tract of COVID-19 patients. Cell Host Microbe.

[10] Wilk AJ (2020). A single-cell atlas of the peripheral immune response in patients with severe COVID-19. Nat. Med..

[11] Lee JS (2020). Immunophenotyping of COVID-19 and influenza highlights the role of type I interferons in development of severe COVID-19. Sci. Immunol..

[12] Schulte-Schrepping J (2020). Severe COVID-19 is marked by a dysregulated myeloid cell compartment. Cell.

[13] Galani I-E (2021). Untuned antiviral immunity in COVID-19 revealed by temporal type I/III interferon patterns and flu comparison. Nat. Immunol..

[14] Wolfel R (2020). Virological assessment of hospitalized patients with COVID-2019. Nature.

[15] Wang N (2020). Retrospective multicenter cohort study shows early interferon therapy is associated with favorable clinical responses in COVID-19 patients. Cell Host Microbe.

[16] Israelow B (2020). Mouse model of SARS-CoV-2 reveals inflammatory role of type I interferon signaling. J. Exp. Med..

[17] Liao M (2020). Single-cell landscape of bronchoalveolar immune cells in patients with COVID-19. Nat. Med..

[18] Ablasser A, Chen ZJ (2019). cGAS in action: expanding roles in immunity and inflammation. Science.

[19] Farber DL (2021). Tissues, not blood, are where immune cells function. Nature.

[20] Freeman EE (2020). The spectrum of COVID-19-associated dermatologic manifestations: an international registry of 716 patients from 31 countries. J. Am. Acad. Dermatol..

[21] Goshua G (2020). Endotheliopathy in COVID-19-associated coagulopathy: evidence from a single-centre, cross-sectional study. Lancet Haematol..

[22] Bonaventura A (2021). Endothelial dysfunction and immunothrombosis as key pathogenic mechanisms in COVID-19. Nat. Rev. Immunol..

[23] Liu S (2015). Phosphorylation of innate immune adaptor proteins MAVS, STING, and TRIF induces IRF3 activation. Science.

[24] Haag SM (2018). Targeting STING with covalent small-molecule inhibitors. Nature.

[25] Berezowska S (2021). Postmortem cardiopulmonary pathology in patients with COVID-19 infection: single-center report of 12 autopsies from Lausanne, Switzerland. Diagnostics.

[26] Varga Z (2020). Endothelial cell infection and endotheliitis in COVID-19. Lancet.

[27] Thacker VV (2021). Rapid endotheliitis and vascular damage characterize SARS-CoV-2 infection in a human lung-on-chip model. EMBO Rep..

[28] Nascimento Conde J, Schutt WR, Gorbunova EE, Mackow ER (2020). Recombinant ACE2 expression is required for SARS-CoV-2 to infect primary human endothelial cells and induce inflammatory and procoagulative responses. mBio.

[29] Kim J (2019). VDAC oligomers form mitochondrial pores to release mtDNA fragments and promote lupus-like disease. Science.

[30] Stukalov A (2021). Multilevel proteomics reveals host perturbations by SARS-CoV-2 and SARS-CoV. Nature.

[31] Gibellini L (2020). Altered bioenergetics and mitochondrial dysfunction of monocytes in patients with COVID-19 pneumonia. EMBO Mol. Med..

[32] Winkler ES (2020). SARS-CoV-2 infection of human ACE2-transgenic mice causes severe lung inflammation and impaired function. Nat. Immunol..

[33] McCray PB (2007). Lethal infection of K18-hACE2 mice infected with severe acute respiratory syndrome coronavirus. J. Virol..

[34] Bao L (2020). The pathogenicity of SARS-CoV-2 in hACE2 transgenic mice. Nature.

[35] Golden JW (2020). Human angiotensin-converting enzyme 2 transgenic mice infected with SARS-CoV-2 develop severe and fatal respiratory disease. JCI Insight.

[36] Muñoz-Fontela C (2020). Animal models for COVID-19. Nature.

[37] Liu Y (2014). Activated STING in a vascular and pulmonary syndrome. N. Engl. J. Med..

[38] Prantner D (2012). 5,6-Dimethylxanthenone-4-acetic acid (DMXAA) activates stimulator of interferon gene (STING)-dependent innate immune pathways and is regulated by mitochondrial membrane potential. J. Biol. Chem..

[39] Neufeldt CJ (2022). SARS-CoV-2 infection induces a pro-inflammatory cytokine response through cGAS-STING and NF-κB. Commun. Biol..

[40] Teuwen L-A, Geldhof V, Pasut A, Carmeliet P (2020). COVID-19: the vasculature unleashed. Nat. Rev. Immunol..

[41] Channappanavar R (2016). Dysregulated type I interferon and inflammatory monocyte–macrophage responses cause lethal pneumonia in SARS-CoV-infected mice. Cell Host Microbe.

[42] Cameron MJ (2007). Interferon-mediated immunopathological events are associated with atypical innate and adaptive immune responses in patients with severe acute respiratory syndrome. J. Virol..

[43] Zhang Q (2020). Inborn errors of type I IFN immunity in patients with life-threatening COVID-19. Science.

[44] Yang DM, Geng TT, Harrison AG, Wang P-H (2021). Differential roles of RIG-I-like receptors in SARS-CoV-2 infection. Military Med. Res..

[45] Mazaleuskaya L, Veltrop R, Ikpeze N, Martin-Garcia J, Navas-Martin S (2012). Protective role of Toll-like receptor 3-induced type I interferon in murine coronavirus infection of macrophages. Viruses.

[46] van der Made CI (2020). Presence of genetic variants among young men with severe COVID-19. JAMA.

[47] Asano, T. et al. X-linked recessive TLR7 deficiency in ~1% of men under 60 years old with life-threatening COVID-19. *Sci. Immunol.* **6**, eabl4348 (2021). 10.1126/sciimmunol.abl4348 PMC8532080 34413140

[48] Xie J (2018). Dampened STING-dependent interferon activation in bats. Cell Host Microbe.

[49] Perkins JR (2012). ReadqPCR and NormqPCR: R packages for the reading, quality checking and normalisation of RT–qPCR quantification cycle (Cq) data. BMC Genomics.

[50] Rappsilber J, Mann M, Ishihama Y (2007). Protocol for micro-purification, enrichment, pre-fractionation and storage of peptides for proteomics using StageTips. Nat. Protoc..

[51] Dorfer V (2014). MS Amanda, a universal identification algorithm optimized for high accuracy tandem mass spectra. J. Proteome Res..

[52] Kong AT, Leprevost FV, Avtonomov DM, Mellacheruvu D, Nesvizhskii AI (2017). MSFragger: ultrafast and comprehensive peptide identification in mass spectrometry–based proteomics. Nat. Methods.

[53] Plubell DL (2017). Extended multiplexing of tandem mass tags (TMT) labeling reveals age and high fat diet specific proteome changes in mouse epididymal adipose tissue. Mol. Cell Proteomics.

[54] Robinson MD, McCarthy DJ, Smyth GK (2010). edgeR: a Bioconductor package for differential expression analysis of digital gene expression data. Bioinformatics.

[55] Ritchie ME (2015). limma powers differential expression analyses for RNA-sequencing and microarray studies. Nucleic Acids Res..

[56] Klipper-Aurbach Y (1995). Mathematical formulae for the prediction of the residual beta cell function during the first two years of disease in children and adolescents with insulin-dependent diabetes mellitus. Med. Hypotheses.

[57] Tai YC, Speed TP (2006). A multivariate empirical Bayes statistic for replicated microarray time course data. Ann. Statist..

