# Supplementary material for: The cGAS–STING pathway drives type I IFN immunopathology in COVID-19
Source: Nature. 2022 Jan 19;603(7899):145–51. doi: 10.1038/s41586-022-04421-w (PMC8891013; doi:10.1038/s41586-022-04421-w)

nature portfolio

https://doi.org/10.1038/s41586-022-04421-w

Supplementary information

The cGAS–STING pathway drives type I IFN immunopathology in COVID-19

In the format provided by the authors and unedited

Supplementary Figure 1

Extended Data Fig 7a. :

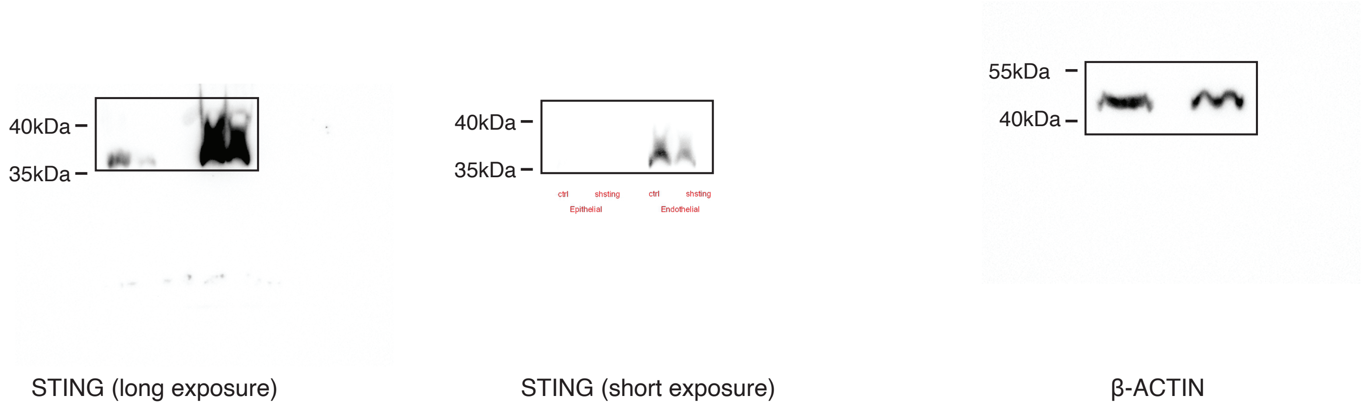

Extended Data Fig 9c. :

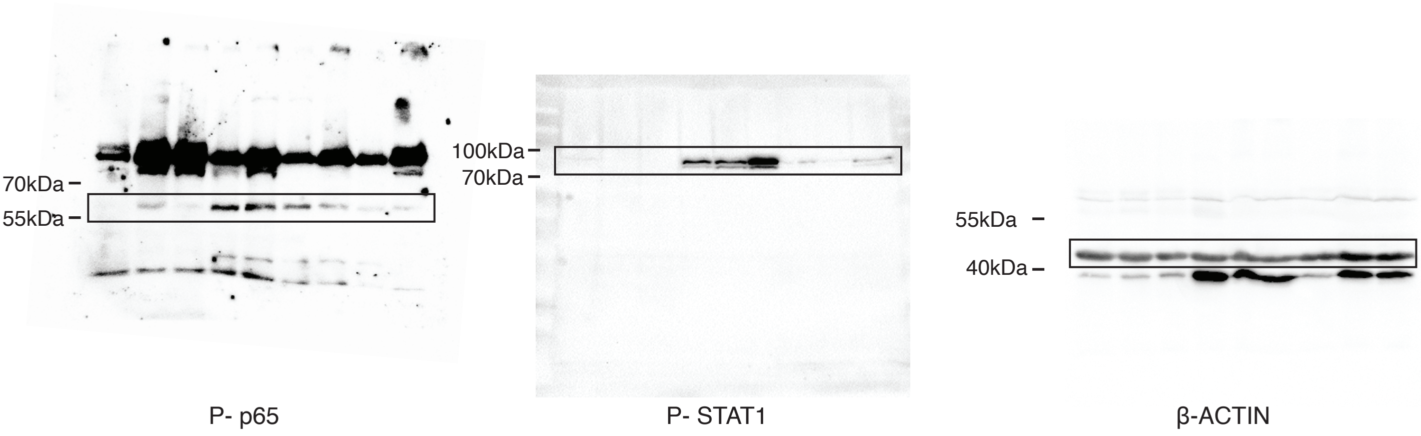

Supplement: Supplementary file 1 — This file contains full scans for all western blots and the in-gel fluorescence images, in Supplementary Fig. 1. [file 41586_2022_4421_MOESM1_ESM.pdf]
