# Supplementary material for: The cGAS–STING pathway drives type I IFN immunopathology in COVID-19
Source: Nature. 2022 Jan 19;603(7899):145–51. doi: 10.1038/s41586-022-04421-w (PMC8891013; doi:10.1038/s41586-022-04421-w)
Supplement: Supplementary file 2 — Reporting Summary [file 41586_2022_4421_MOESM2_ESM.pdf]

nature research

Corresponding author(s): Michel Gilliet, Andrea Ablasser

Last updated by author(s): Jan 5, 2022

Reporting Summary

Nature Research wishes to improve the reproducibility of the work that we publish. This form provides structure for consistency and transparency in reporting. For further information on Nature Research policies, see our Editorial Policies and the Editorial Policy Checklist.

Statistics

For all statistical analyses, confirm that the following items are present in the figure legend, table legend, main text, or Methods section.

n/a	Confirmed	
☐	☒	The exact sample size (*n*) for each experimental group/condition, given as a discrete number and unit of measurement
☐	☒	A statement on whether measurements were taken from distinct samples or whether the same sample was measured repeatedly
☐	☒	The statistical test(s) used AND whether they are one- or two-sided<br>*Only common tests should be described solely by name; describe more complex techniques in the Methods section.*
☒	☐	A description of all covariates tested
☐	☒	A description of any assumptions or corrections, such as tests of normality and adjustment for multiple comparisons
☐	☒	A full description of the statistical parameters including central tendency (e.g. means) or other basic estimates (e.g. regression coefficient) AND variation (e.g. standard deviation) or associated estimates of uncertainty (e.g. confidence intervals)
☐	☒	For null hypothesis testing, the test statistic (e.g. *F*, *t*, *r*) with confidence intervals, effect sizes, degrees of freedom and *P* value noted<br>*Give P values as exact values whenever suitable.*
☒	☐	For Bayesian analysis, information on the choice of priors and Markov chain Monte Carlo settings
☒	☐	For hierarchical and complex designs, identification of the appropriate level for tests and full reporting of outcomes
☒	☐	Estimates of effect sizes (e.g. Cohen's *d*, Pearson's *r*), indicating how they were calculated

Our web collection on statistics for biologists contains articles on many of the points above.

Software and code

Policy information about availability of computer code

Data collection	Human Samples: Data were collected in part with nCounter® Systems, CaseCenter™ slide management system, ZEN 3.2 Imaging Software, SkanIt™ Software, WinTEM software<br><br>For Loc studies: Leica Application Suite Advanced Fluorescence for the Leica SP8 systems. T-qPCR data were acquired using the ABI Systems 7900HT Real-Time PCR system (Applied Biosciences)<br><br>For the rest: Western blots were acquired using Image Lab software (Biorad). RT-qPCR data were acquired using QuantStudio (5, 6 or 7) Real-Time PCR system (ThermoFisher). Microscopic images were aquired using Axiovision software using Axioplan (Zeiss), Olympus OlyVIA software using Olympus V120 whole slide scanner. Western Blot images were analysed using Image Lab (Biorad). ECL signal was recorded using ChemiDoc XRS Biorad Imager.
Data analysis	For Human Samples: Data were anlyzed using nSolver™ Analysis Software, ROSALIND® by OnRamp Bio, QuantCenter plugin 2.2 of Caseviewer 2.4 software, ZEN 3.2 Imaging Software, ImageJ 1.53c software, GraphPad PRISM 8, Microsoft Excel<br><br>Images in Loc studies were analysed and rendered using FIJI with Image J 1.53, and Bitplane Imaris 9.7.2. Data on proteomic networks from String DB was visualised using Cytoscape (3.8.2), and heatmaps generated using Heatmapper. 3D reconstructions of mitochondria were rendered using Blender 2.9.

For manuscripts utilizing custom algorithms or software that are central to the research but not yet described in published literature, software must be made available to editors and reviewers. We strongly encourage code deposition in a community repository (e.g. GitHub). See the Nature Research guidelines for submitting code & software for further information.

Data

Policy information about availability of data

All manuscripts must include a data availability statement. This statement should provide the following information, where applicable:

- Accession codes, unique identifiers, or web links for publicly available datasets
- A list of figures that have associated raw data
- A description of any restrictions on data availability

Full scans for all western blots and the in-gel fluorescence images, are provided in Supplementary Fig. 1 and the Limma and Timecourse analysis of the proteomics data is provided in Supplementary Table 3. Source data for each figure are provided in the corresponding "Source Data" files. Raw data supporting the findings of this study are deposited at Zenodo and will be publicly available at doi:10.5281/zenodo.5818157 [available upon publication]. The proteomics dataset generated during this study is deposited in PRIDE with accession code [available during proof stage]. The genomics dataset generated during this study is deposited at GEO with accession code [available during proof stage].

Field-specific reporting

Please select the one below that is the best fit for your research. If you are not sure, read the appropriate sections before making your selection.

☒ Life sciences ☐ Behavioural & social sciences ☐ Ecological, evolutionary & environmental sciences

For a reference copy of the document with all sections, see nature.com/documents/nr-reporting-summary-flat.pdf

Life sciences study design

All studies must disclose on these points even when the disclosure is negative.

Sample size	We have used at least three biological replicates for each experiment - unless stated otherwise. This is consistent with previous studies and accounts for biological variability in between distinct samples from inbred mice or from cell lines. The number of the mice used for the study was estimated based on the published studies related to this animal model. (n= 5 - 8 mice for histopathological studies and n= 14-15 mice for the survival study. PMID: 32380511, PMID: 33257679)
Data exclusions	No data was excluded.
Replication	Experimental findings were reliably reproduced. The number (n) of biological replicates or animals is indicated as an exact number in the figure legends.
Randomization	In LoC experiments, for comparisons between different experimental conditions, fields of view ca. 232 x 232 x 10 µm3 were chosen randomly from the epithelial and endothelial layers after a scan through the entire chip.<br><br>Mice were randomly allocated to distinct groups
Blinding	LoC experiments: blinding was not possible as experimental conditions were evident from image data.<br>In vivo studies: Experimenters were blinded for the analysis of the histopathological scores for experimental groups.

Reporting for specific materials, systems and methods

We require information from authors about some types of materials, experimental systems and methods used in many studies. Here, indicate whether each material, system or method listed is relevant to your study. If you are not sure if a list item applies to your research, read the appropriate section before selecting a response.

Materials & experimental systems

n/a	Involved in the study	
☐	☒	Antibodies
☐	☒	Eukaryotic cell lines
☒	☐	Palaeontology and archaeology
☐	☒	Animals and other organisms
☐	☒	Human research participants
☒	☐	Clinical data
☒	☐	Dual use research of concern

Methods

n/a	Involved in the study	
☒	☐	ChIP-seq
☒	☐	Flow cytometry
☒	☐	MRI-based neuroimaging

Antibodies

Antibodies used	Primary antibodies:<br>β-actin-HRP (C4, Santa Cruz 1:5000 dilution WB)

	Rabbit anti-Phospho-Stat1 (D4A7, Cell Signaling 1:1000 WB)<br>Rabbit anti-Phospho-p65 (S468) (Cell Signaling 1:1000 WB)<br>Mouse anti-human CD31 (P2B1, monoclonal, Abcam ab24590)<br>Mouse anti-human CD45 (MEM-28, monoclonal, Abcam ab8216)<br>Mouse anti-human CD45 (2D1, monoclonal, BioLegend 368537)<br>Mouse anti-human CD163 (10D6, DIAGNOSTIC BIOSYSTEMS, 1:50)<br>Rabbit anti-human CD163 (Cat# LS-A10716, LSBio, 1:400)<br>Mouse anti-human CD123 (7G3, BD biosciences, 1:200)<br>Rabbit anti-human Myeloperoxidase (Cat# A0398, DAKO, 1:1000)<br>Rabbit anti-human CD3 (2GV6, VENTANA, Ready to use)<br>Mouse anti-human CD31 (JC70A, DAKO, 1:100)<br>Rabbit anti-human IFN-beta (Cat# PA5-20390, ThermoFisherScientific, 1:1000)<br>Rabbit anti-human Cleaved Caspase-3 (Asp175) (Cat #9661, Cell Signaling Technology, 1:200)<br>Rabbit anti-human Phospho-STING (Ser366) (D7C3S, Cell Signaling Technology, 1:100)<br>Goat anti-human Mx1/2/3 (D-14, Santa Cruz, 1:50)<br><br>Secondary antibodies:<br>Donkey anti-rabbit IgG (H+L) AF546 (A10040, ThermoFisher, 1:500),<br>Goat anti-Rabbit IgG (H+L) AF546 (A11035, ThermoFisher, 1:500),<br>Goat anti-mouse IgG1 AF488 (A21121, ThermoFisher, 1:500),<br>Donkey anti-rabbit IgG (H+L) AF488 (A32790, ThermoFisher, 1:500),<br>Donkey Anti-Goat IgG H&L (HRP) (ab97110, Abcam, 1:500),<br>Goat anti-mouse Alexa Fluor 488 (A-11029, Thermo Fisher),<br>Donkey anti-mouse Alexa Fluor 568 (A-10037, Thermo Fisher),<br>Donkey anti-rabbit Alexa Fluor 488 (A-21206, Thermo Fisher),<br>Donkey anti-rabbit Alexa Fluor 568 (A-10042, Thermo Fisher),
Validation	Primary Antibodies were validated for use for immunofluorescence for human or mouse respectively by the manufacturers as stated on their respective websites. Aliquots of secondary antibodies were provided by the Histology Core Facility at EPFL and have been validated by the manufacturers.<br><br>For patient samples:<br>Mouse anti-human CD163: IHC<br>Rabbit anti-human CD163: IHC<br>Mouse anti-human CD123: IF, IP, WB, Blocking, Neutralization<br>Rabbit anti-human Myeloperoxidase: IHC<br>Rabbit anti-human CD3: IHC<br>Mouse anti-human CD31: IHC<br>Rabbit anti-human IFN-beta: WB, IHC, IF, F, Elisa<br>Rabbit anti-human Cleaved Caspase-3 (Asp175): WB, IP , IHC, IF, F<br>Rabbit anti-human Phospho-STING (Ser366): WB<br>Goat anti-human Mx1/2/3: WB, IP, IF, Elisa

Eukaryotic cell lines

Policy information about cell lines

Cell line source(s)	Primary human alveolar epithelial cells and human lung microvascular endothelial cells were purchased from Cell Biologics, USA via PeloBiosciences in Switzerland. WT and cGAS-/- THP-1 cells were purchased from Invivogen. Vero-E6 cells were a kind gift from the lab of Prof Carolyn Tapparel at the University of Geneva and HEK-293T cells were a gift from the lab of Prof Didier Trono at EPFL.
Authentication	The identity of primary human cells and of the THP-1 cell lines were verified by the supplier Cell Biologics and Invivogen respectively.
Mycoplasma contamination	All primary cells used without passage were verified to be mycoplasma free by the supplier. Passaged lung microvascular endothelial cells, THP-1 , Vero-E6, and HEK-293T cell lines were verified to be mycoplasma free at EPFL.
Commonly misidentified lines<br>(See ICLAC register)	none

Animals and other organisms

Policy information about studies involving animals; ARRIVE guidelines recommended for reporting animal research

Laboratory animals	In this study, the following mouse strain was used B6.Cg-Tg(K18-ACE2)2Prlmn/J (https://www.jax.org/strain/034860). For viral challenge female eight-week old mice were challenged intranasally with SARS-CoV-2.<br><br>Mice were housed in groups of up to 5 mice/cage at 18 degrees C-24 degrees C ambient temperatures with 40-60% humidity. Mice were maintained on a 12 hour light/ dark cycle 6 am to 6 pm. Food and water were available ad libitum.
Wild animals	The study did not involve wild animals

Field-collected samples

The study did not involve samples collected from the field

Ethics oversight

Animal experiments were approved by the Service de la Consommation et des Affaires Vétérinaires of the canton of Vaud (Switzerland) and were performed in accordance with the respective legal regulations.

Note that full information on the approval of the study protocol must also be provided in the manuscript.

Human research participants

Policy information about studies involving human research participants

Population characteristics

Patient_code gender age diagnosis
HD-01 M 48 healthy donor
HD-02 M 55 healthy donor
HD-03 M 30 healthy donor
HD-04 M 44 healthy donor
PV-01 M 21 plaque-type psoriasis
PV-02 M 44 plaque-type psoriasis
PV-03 M 66 plaque-type psoriasis
PV-04 M 49 plaque-type psoriasis
PV-05 M 41 plaque-type psoriasis
PV-06 F 47 plaque-type psoriasis
PV-07 M 57 plaque-type psoriasis
PV-08 M 43 plaque-type psoriasis
PV-09 M 29 plaque-type psoriasis
PV-10 M 32 plaque-type psoriasis
PV-11 M 34 plaque-type psoriasis
PV-12 M 33 plaque-type psoriasis
PV-13 F 37 plaque-type psoriasis
PV-14 M 66 plaque-type psoriasis
PV-15 M 39 plaque-type psoriasis
PV-16 F 48 plaque-type psoriasis
PV-17 F 55 plaque-type psoriasis
PV-18 M 63 plaque-type psoriasis
PV-19 M 48 plaque-type psoriasis
PV-20 M 58 plaque-type psoriasis
PV-21 M 36 plaque-type psoriasis
AD-01 M 33 atopic dermatitis
AD-02 M 32 atopic dermatitis
AD-03 F 23 atopic dermatitis
AD-04 M 39 atopic dermatitis
AD-05 M 31 atopic dermatitis
AD-06 F 22 atopic dermatitis
AD-07 M 53 atopic dermatitis
AD-08 M 28 atopic dermatitis
AD-09 F 29 atopic dermatitis
AD-10 M 25 atopic dermatitis
AD-11 M 82 atopic dermatitis
AD-12 M 27 atopic dermatitis
AD-13 M 44 atopic dermatitis
AD-14 M 27 atopic dermatitis
AD-15 M 61 atopic dermatitis
AD-16 M 46 atopic dermatitis
LP-01 M 33 lichen planus
LP-02 F 54 lichen planus
LP-03 M 39 lichen planus
LP-04 F 47 lichen planus
LP-05 M 57 lichen planus
CLE-01 F 40 cutaneous lupus erythematosus
CLE-02 M 40 cutaneous lupus erythematosus
CLE-03 F 52 cutaneous lupus erythematosus
CLE-04 F 47 cutaneous lupus erythematosus
CLE-05 F 31 cutaneous lupus erythematosus
CLE-06 M 78 cutaneous lupus erythematosus
CLE-07 M 46 cutaneous lupus erythematosus
CLE-08 M 36 cutaneous lupus erythematosus
CLE-09 F 64 cutaneous lupus erythematosus
CLE-10 F 46 cutaneous lupus erythematosus
CLE-11 F 27 cutaneous lupus erythematosus
CLE-12 F 21 cutaneous lupus erythematosus
CLE-13 F 67 cutaneous lupus erythematosus
CLE-14 F 52 cutaneous lupus erythematosus
CLE-15 F 25 cutaneous lupus erythematosus
CLE-16 F 50 cutaneous lupus erythematosus
COVID-01 F 62 COVID-19-associated skin lesion

COVID-02 F 65 COVID-19-associated skin lesion
COVID-03 F 34 COVID-19-associated skin lesion
COVID-04 F 21 COVID-19-associated skin lesion
COVID-05 F 62 COVID-19-associated skin lesion
COVID-06 M 47 COVID-19-associated skin lesion
COVID-07 M 18 COVID-19-associated skin lesion
COVID-08 M 22 COVID-19-associated skin lesion
COVID-09 M 56 COVID-19-associated skin lesion
COVID-10 M 80 COVID-19-associated skin lesion

Recruitment

Residual skin materials were obtained from the Dermatology Biobank at CHUV from patients with COVID-19-associated skin lesions, cutaneous lupus erythematosus (CLE), lichen planus (LP), atopic dermatitis (AD), and plaque-type psoriasis (PV). For healthy controls, residual healthy skin was obtained from surgery.

Ethics oversight

Studies were approved by the University Hospital of Lausanne (CHUV) and were performed in accordance with the guidelines of the Declaration of Helsinki and were reviewed by the ethical committee board of the canton of Vaud (CER-VD 2020-02204).

Note that full information on the approval of the study protocol must also be provided in the manuscript.
